# Ferrocenyl–triazole complexes and their use in heavy metal cation sensing†

**DOI:** 10.1039/d4ra04023f

**Published:** 2024-06-28

**Authors:** Khaled Al Khalyfeh, Asma Ghazzy, Randa M. Al-As' ad, Tobias Rüffer, Olfa Kanoun, Heinrich Lang

**Affiliations:** a Department of Chemistry, Faculty of Natural Science, Al-Hussein Bin Talal University Ma'an 71111 Jordan k.khalyfeh@ahu.edu.jo; b Faculty of Pharmacy, Faculty of Pharmacy and Applied Medical Sciences, Al-Ahliyya Amman University Amman 19328 Jordan; c Pharmacological and Diagnostic Research Center, Faculty of Pharmacy and Allied Medical Sciences, Al-Ahliyya Amman University Amman 19328 Jordan; d Department of Inorganic Chemistry, Chemnitz University of Technology 09111 Chemnitz Germany; e Professorship Measurement and Sensor Technology, Chemnitz University of Technology 09126 Chemnitz Germany; f Research Center for Materials, Architectures and Integration of Nanomembranes (MAIN), Research Group Organometallics, Chemnitz University of Technology 09126 Chemnitz Germany

## Abstract

Complexes tris((1-ferrocenyl-1*H*-1,2,3-triazol-4-yl)methyl)amine (3), bis((1-ferrocenyl-1*H*-1,2,3-triazol-4-yl)methyl)amine (6), bis((1-ferrocenyl-1*H*-1,2,3-triazol-4-yl)methyl)ether (7), and 1-ferrocenyl-1*H*-1,2,3-triazol-4-yl)methanamine (9) were synthesized using the copper-catalyzed click reaction. Complexes 3, 6, 7, and 9 were characterized using NMR (^1^H and ^13^{^1^H}) and IR spectroscopy, elemental analysis, and mass spectrometry. Structures of 3, 7, and 9 in the solid state were determined using single-crystal X-ray diffraction. It was found that the triazole rings were planar and slightly twisted with respect to the cyclopentadienyl groups attached to them. Chains and 3D network structures were observed due to the presence of π⋯π and C–H⋯N interactions between the cyclopentadienyl and triazole ligands. A reversible redox behavior of the Fc groups between 239 and 257 mV with multicycle stability was characteristic for all the compounds, revealing that the electrochemically generated species Fc^+^ remained soluble in dichloromethane. Electrochemical sensor tests demonstrated the applicability of all the complexes to enhance the quantification sensing behavior of the screen-printed carbon electrode (SPCE) toward Cd^2+^, Pb^2+^, and Cu^2+^ ions.

## Abstract 2

Ferrocenyl–triazoles synthesized, characterized and electrochemically investigated. They uses as modifier for the screen printed carbon electrodes proven toward cations sensing.

## Graphical abstract

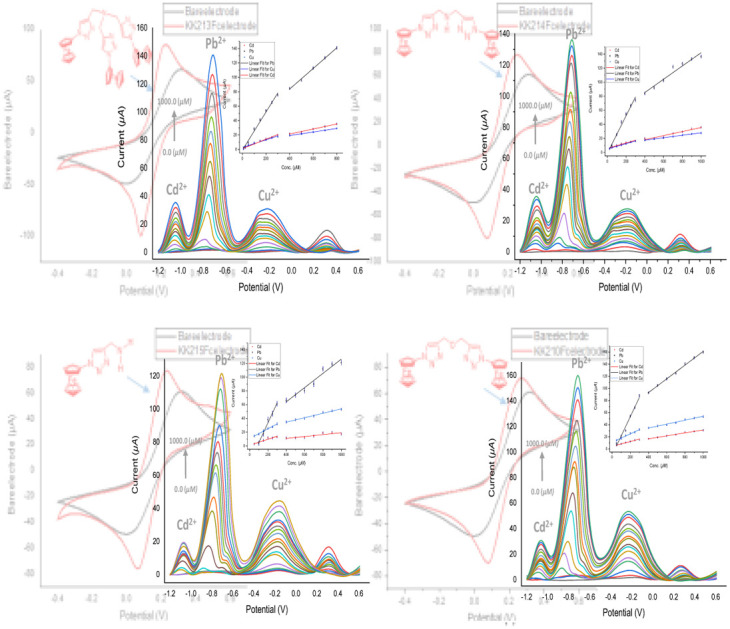

## 1. Introduction

Due to the limited resources of water around the world, waste-water treatment processes have received much attention from researchers, especially towards heavy metals, which are significantly discharged with the rapid development of industry and have now become one of the main components responsible for environmental pollution.^1–5^ Heavy metals are non-biodegradable and hence can accumulate during the living cycle, even at a trace level in living tissues,^6–8^ whereby they can cause several diseases as well as affect the living organs of humans as well as other organisms.^9–11^ The detection of such metals, for example, using state-of-the-art techniques, including atomic-fluorescence and inductively coupled plasma mass spectrometry, is expensive and requires high operating costs (*e.g.*, for sample preparation, training operators, time).^12,13^

Recently, chemo-sensing portable devices with advantages of simple, rapid, sensitive, and selective operation are still lacking. Modifying the surface of electrochemical sensor electrodes has become the key element in today's diverse sensing technologies.^4,5,14–17^ Among the electrochemical sensor electrodes, modified screen-printed carbon electrodes (SPCEs) due to their unique advantages such as facile mass production, disposable cast, low cost, reproducibility and sensitivity are widely applied to construct highly selective electrodes for electrochemical detection in environmental applications.^18–20^ In recent advances in electrode modification, inorganic materials/nanomaterials are often used.^4,5,21–23^ However, simple, inexpensive, and reversible redox-active modifying materials for signal amplification in electrochemical sensors are still lacking.^24–28^ The unique redox activity of ferrocene and some of its derivatives have raised their potential as chemo-sensing materials.^29–33^ In addition, heterocyclic compounds such as oxazolines, imidazoles, and triazoles have proven to be efficient recognition units for metal cation detection.^34–39^ Therefore, ferrocenes are considered invaluable templates as redox-moieties and in combination with a triazole-heterocyclic derivative can be expected to offer a greater surface area available for the deposition of specific analytes as well as an enhancement in the electron-transfer processes, and consequently, such compounds should stimulate the modified electrodes to behave as multiple signaling molecular selective chemo-sensors for metal cations.^4,5,29,40–48^

Herein, we present the synthesis and characterization of diverse ferrocenyl-containing triazole complexes. In addition, their structural and electrochemical properties are reported. Their use as modifier materials to design a voltammetric sensor for the quantification of heavy metals is discussed.

## 2. Experimental

### 2.1. General data

All the reactions were carried out under an argon atmosphere (5.0) employing standard Schlenk techniques. Tetrahydrofuran and *N*,*N*-dimethylformamide were purified by distillation from sodium/benzophenone and calcium hydride, respectively. For the electrochemistry experiments, HPLC grade dichloromethane was purified by distillation from calcium hydride. For the column chromatography, alumina with a particle size of 90 μm (Standard, Merck KGaA) and silica with a particle size of 40–60 μm (230–400 mesh (ASTM), Fa. Macherey-Nagel) were used.

### 2.2. Instruments

A Bruker Avance III 500 spectrometer operating in the Fourier transform mode at 298 K was utilized to acquire the NMR spectra, including (^1^H NMR (500.3 MHz) and ^13^C{^1^H} NMR (125.8 MHz)). Chemical shifts (*δ*) are reported in parts per million (ppm) relative to tetramethylsilane using the solvent as the internal reference (CDCl_3_: ^1^H NMR *δ* = 7.26 ppm; ^13^C{^1^H} NMR *δ* = 77.16 ppm).^49^ A FT-Nicolet IR 200 spectrometer was employed to capture the infrared spectra. The determination of the melting points utilized analytical pure samples tested with a Gallenkamp MFB 595 010 M melting point apparatus.

Elemental analyses were conducted using a Thermo FlashEA 1112 Series instrument, and high-resolution mass spectra were recorded using a micrOTOF QII Bruker Daltonite spectrometer.

### 2.3. Reagents

All the starting materials were obtained from commercial suppliers and used without further purification. [N^*n*^Bu_4_][B(C_6_F_5_)_4_],^50,51^ and 1-azidoferrocene (1)^52,53^ were synthesized according to published procedures.

### 2.4. Synthesis

#### 2.4.1. General procedure for the classical and enhanced click chemistry experiments

1-Azidoferrocene (1) (1 eq.), tripropargylamine (2) (0.3 eq. for 3); dipropargylamine (4) (0.4 eq. for 6); dipropargylether (5) (0.4 eq. for 7); propargylamine (8) (0.9 eq. for 9), and tris((1-ferrocenyl-1*H*-1,2,3-triazol-4-yl)methyl)amine (3) ((0.02 eq. for 6 and 7) and (0.05 eq. for 9)) were dissolved in a mixture of anhydrous tetrahydrofuran (60 mL) and deionized water (10 mL). Sequentially, copper sulfate pentahydrate (0.09 eq.) and sodium ascorbate (0.9 eq.) dissolved in 20 mL of deionized water were added dropwise. After stirring the reaction mixture at 25 °C for 48 h, all the volatiles were evaporated in a vacuum. Afterward, 100 mL of dichloromethane was added in a single portion. The organic layer was washed with aqueous ammonium hydroxide until a colorless aqueous layer was obtained and then with water to neutrality. The organic phase was dried (MgSO_4_) and all the volatiles were removed using an oil pump vacuum. Column chromatography of the crude product (100 × 15 mm silica, eluent: dichloromethane, acetonitrile, and then acetonitrile/dimethylformamide 3/1 (v/v)) was carried out.

#### 2.4.2. Synthesis of tris((1-ferrocenyl-1*H*-1,2,3-triazol-4-yl)methyl)amine (3)

Reagents: 1-Azidoferrocene (1) (1.00 g, 4.41 mmol), tripropargylamine (2) (0.18 g, 1.33 mmol), copper sulfate pentahydrate (0.10 g, 400.2 μmol), and sodium ascorbate (0.79 g, 4.00 mmol). Product 3: 1.02 g, 94% (based on 2) was obtained as a brown solid; elemental analysis calcd for C_39_H_36_Fe_3_N_10_ (812.32 g mol^−1^): C, 57.67; H, 4.47; N, 17.24 found: C, 57.59; H, 4.53; N, 17.29; Mp: 153.0 ^13^C; ^1^H-NMR (CDCl_3,_*δ*/ppm): 8.09 (s, 3H, -^*N*^C_2_HN_3_), 4.89 (t, *J* = 2.0 Hz, 6H, C_5_H_4_), 4.28 (t, *J* = 2.0 Hz, 6H, C_5_H_4_), 4.23 (s, 15H, C_5_H_5_), 3.87 (s, 6H, CH_2_); ^13^C-NMR (CDCl_3,_*δ*/ppm): 143.9 (C, -^*N*^C_2_HN_3_), 123.8 (CH, -^*N*^C_2_HN_3_), 93.9 (^*i*^C, –C_5_H_4_), 70.3 (C–C_5_H_4_), 66.8 (C–C_5_H_4_), 62.2 (C–C_5_H_5_), 47.1 (C, (–CH_2_)_3_–N); IR (NaCl, cm^−1^): 3091 (w), 1520 (s), 1218 (m), 1107 (s), 1044 (m), 879 (m), 816 (s); HRMS (ESI-TOF, *m*/*z*): calcd 835.1065, found: 835.1050 [M]^+^.

#### 2.4.3. Synthesis of bis((1-ferrocenyl-1*H*-1,2,3-triazol-4-yl)methyl)amine (6)

Reagents: 1-Azidoferrocene (1) (1.00 g, 4.41 mmol), dipropargylamine (4) (0.17 g, 1.77 mmol), and tris((1-ferrocenyl-1*H*-1,2,3-triazol-4-yl)methyl)amine (3) (0.07 g, 88.6 μmol), copper sulfate pentahydrate (0.10 g, 400.2 μmol), and sodium ascorbate (0.79 g, 4.00 mmol). Product 6: 0.85 g, 88% (based on 4) was obtained as a brownish solid; elemental analysis calcd for C_26_H_25_Fe_2_N_7_ (547.23 g mol^−1^): C, 57.07; H, 4.61; N, 17.92. Found: C, 56.99; H, 4.68; N, 17.85; Mp: 146.0 °C; ^1^H-NMR (CDCl_3,_*δ*/ppm): 7.78 (s, 2H, -^*N*^C_2_HN_3_), 4.82 (t, *J* = 1.9 Hz, 4H, C_5_H_4_), 4.26 (t, *J* = 1.9 Hz, 4H, C_5_H_4_), 4.21 (s, 10H, C_5_H_5_), 4.02 (s, 4H, CH_2_), 2.04 (s, 1H, NH); ^13^C-NMR (CDCl_3,_*δ*/ppm): 146.3 (C, -^*N*^C_2_HN_3_), 121.8 (CH, -^*N*^C_2_HN_3_), 93.9 (^*i*^C, –C_5_H_4_), 70.3 (C–C_5_H_4_), 66.8 (C–C_5_H_4_), 62.2 (C–C_5_H_5_), 43.9 (C, (–CH_2_)_2_–NH); IR (NaCl, cm^−1^): 3290 (m), 3124 (s), 3093 (s), 2924 (w), 1516 (s), 1410 (m), 1220 (s), 1106 (s), 1042 (s), 1024 (s), 1000 (s), 818 (s); HRMS (ESI-TOF, *m*/*z*): calcd 570.0762, found: 570.0753 [M]^+^.

#### 2.4.4. Synthesis of bis((1-ferrocenyl-1*H*-1,2,3-triazol-4-yl)methyl)ether (7)

Reagents: 1-Azidoferrocene (1) (1.00 g, 4.41 mmol), dipropargylether (5) (0.17 g, 1.76 mmol), tris((1-ferrocenyl-1*H*-1,2,3-triazol-4-yl)methyl)amine (3) (0.07 g, 88.6 μmol), copper sulfate pentahydrate (0.10 g, 400.2 μmol), and sodium ascorbate (0.79 g, 4.00 mmol). Product 7: 0.86 g, 89% (based on 5) was obtained as a brownish solid; elemental analysis calcd for C_26_H_24_Fe_2_N_6_O (548.21 g mol^−1^): C, 56.96; H, 4.41; N, 15.33. Found: C, 57.11; H, 4.47; N, 15.39; Mp: 152.5 ^13^C; ^1^H-NMR (CDCl_3,_*δ*/ppm): 7.85 (s, 2H, -^*N*^C_2_HN_3_), 4.83 (t, *J* = 1.9 Hz, 4H, C_5_H_4_), 4.79 (s, 4H, CH_2_), 4.27 (t, *J* = 1.9 Hz, 4H, C_5_H_4_), 4.22 (s, 10H, C_5_H_5_); ^13^C-NMR (CDCl_3,_*δ*/ppm): 144.6 (C, -^*N*^C_2_HN_3_), 122.8 (CH, -^*N*^C_2_HN_3_), 93.9 (^*i*^C, –C_5_H_4_), 70.3 (C–C_5_H_4_), 66.9 (C–C_5_H_4_), 63.8 (C–C_5_H_5_), 62.3 (C, (–CH_2_)_2_–O); IR (NaCl, cm^−1^): 3126 (m), 3092 (m), 2861 (m), 1517 (s), 1410 (m), 1368 (m), 1222 (s), 1106 (s), 1097 (s), 1042 (s), 1000 (s), 878 (s), 823 (s); HRMS (ESI-TOF, *m*/*z*): calcd 571.0603, found: 571.0592 [M]^+^.

#### 2.4.5. Synthesis of (1-ferrocenyl-1*H*-1,2,3-triazol-4-yl)methanamine (9)

Reagents: 1-Azidoferrocene (1) (1.00 g, 4.41 mmol), propargylamine (8) (0.22 g, 4.01 mmol), tris((1-ferrocenyl-1*H*-1,2,3-triazol-4-yl)methyl)amine (3) (0.18 g, 220.2 μmol), copper sulfate pentahydrate (0.10 g, 400.2 μmol), and sodium ascorbate (0.79 g, 4.00 mmol). Product 9: 0.96 g, 92% (based on 8) was obtained as a yellow solid; elemental analysis calcd for C_13_H_14_FeN_4_ (282.13 g mol^−1^): C, 55.34; H, 5.00; N, 19.86. Found: C, 55.18; H, 5.12; N, 19.79; Mp: 101.5 °C; ^1^H-NMR (CDCl_3,_*δ*/ppm): 7.69 (s, 1H, -^*N*^C_2_HN_3_), 4.82 (t, *J* = 2.0 Hz, 2H, C_5_H_4_), 4.26 (t, *J* = 2.0 Hz, 2H, C_5_H_4_), 4.21 (s, 5H, C_5_H_5_), 4.04 (s, 2H, CH_2_), 1.57 (s, 2H, NH_2_); ^13^C-NMR (CDCl_3,_*δ*/ppm): 146.2 (C, -^*N*^C_2_HN_3_), 121.7 (CH, -^*N*^C_2_HN_3_), 93.8 (^*i*^C, –C_5_H_4_), 70.2 (C–C_5_H_4_), 66.7 (C–C_5_H_4_), 62.1 (C–C_5_H_5_), 37.8 (C,–CH_2_–NH_2_); IR (NaCl, cm^−1^): 3454 (m), 3138 (m), 3095 (m), 2925 (m), 1520 (s), 1213 (s), 1106 (s), 1046 (s), 1027 (s), 1000 (s), 818 (s); HRMS (ESI-TOF, *m*/*z*): calcd 283.0641, found: 283.0637 [H]^+^.

### 2.5. Electrochemistry

Electrochemical measurements were performed using 1.0 mmol L^−1^ solutions of the analytes and [N^*n*^Bu_4_][B(C_6_F_5_)_4_] as the supporting electrolyte in anhydrous dichloromethane at 25 °C.^32,33,38,54^ The instrumentation consisted of a Radiometer Gamry interface 1010E workstation interfaced with a personal computer. The measurement cell comprised three electrodes: a Pt auxiliary electrode, a glassy carbon working electrode, and a Ag/Ag^+^ (0.01 mol L^−1^ AgNO_3_) reference electrode. The working electrode underwent pretreatment involving polishing on a Buehler microcloth subsequently with 1 μm and 1/4 μm diamond paste. The reference electrode composed a silver wire, which was inserted into a Luggin capillary with a Vycor tip filled with a solution of 0.01 mol L^−1^ [AgNO_3_] and 0.1 mol L^−1^ [N^*n*^Bu_4_][B(C_6_F_5_)_4_] in acetonitrile. This Luggin capillary was further inserted into a second Luggin capillary with a Vycor tip filled with a solution of 0.1 mol L^−1^ [N^*n*^Bu_4_][B(C_6_F_5_)_4_] in dichloromethane.^54–60^ Under these conditions, all the experiments demonstrated that all the oxidation and reduction processes were reproducible in the range of ±5 mV. The experimental potentials were internally referenced against a Ag/Ag^+^ reference electrode, while all the presented results are referenced against ferrocene (as internal standard) as recommended by IUPAC.^61^ The experimentally measured potential was adjusted into *E vs.* FcH/FcH^+^ by adding −614 mV. This correction was applied when decamethylferrocene served as an internal standard. Under our experimental conditions, the Fc*/Fc*^+^ couple was at −614 mV *vs.* FcH/FcH^+^, Δ*E*_p_ = 60 mV, and the FcH/FcH^+^ couple itself was at 220 mV *vs.* Ag/Ag^+^, (Δ*E*_p_ = 61 mV).^62–64^ A Microsoft Excel worksheet was employed to process the data, ensuring the formal redox potentials of the FcH/FcH^+^ couple were set to *E*° = 0.0 V.^62^ The cyclic voltammograms were obtained after two scans and were deemed to be steady-state cyclic voltammograms, wherein the signal pattern remained consistent with the initial sweep.

### 2.6. Electrochemical sensing

The sensing measurements were conducted with a PalmSens4 portable potentiostat (Palmsens BV, GA Houten, Netherlands) and screen-printed carbon electrodes (SPCEs) featuring a carbon counter electrode, a graphite working electrode, and a Ag/AgCl reference electrode.^65^ The selection of an appropriate buffer as an electrolyte for metal analysis was necessary to avoid the metal cations precipitation.^66^ Therefore, HAcO–NaAcO buffer solution (ABS (0.1 M), pH = 5.6) was used as the supporting electrolyte. The electrochemical responses for ABS (0.1 M, pH = 5.6) containing the target cations were recorded by square wave voltammetry measurements in the potential range from –1.3 V to + 1.0 V, modulation amplitude of 50 mV, step potential of ±5 mV, and equilibration time of 5 s.

### 2.7. Functionalization of the SPCEs with the ferrocenyl–triazole complexes

To improve the performance of the SPCEs, additives may be used to enhance the response behaviors, sensitivity, and selectivity.^22,67–69^ Thus, dichloromethane solutions (1.0 mM) of the ferrocenyl–triazole complexes 3, 6, 7, and 9 were prepared and used to modify the working SPCEs using the drop-casting technique with the desired complex solution (1 μL × 5 times) and then dried at room temperature and atmospheric pressure for approximately 6 h.

### 2.8. Single-crystal X-ray diffraction analysis

The diffusion of pentane into a dichloromethane solution containing 3, 7, or 9 at ambient temperature offered suitable single crystals for X-ray diffraction analysis. An Oxford Gemini S diffractometer (3, Mo Kα radiation, *λ* = 0.71073 Å, 120 K) and a Bruker Venture D8 diffractometer (7/9, Cu Kα radiation, *λ* = 1.54178 Å, 100 K) were utilized to acquire the corresponding data. The molecular structures were determined through direct methods and refined using full-matrix least-squares procedures on *F*_o_^2^ using SHELXS- and SHELXL-2013 implemented in the WINGX v2013.3 suite.^70,71^ All non-hydrogen atoms were refined anisotropically, and a riding model was employed for treatment of the hydrogen atom positions.

In the case of 7, the data set was treated with the command SQUEEZE of the PLATON program.^72^ Within the unit cell (*V* = 2650.4(2) Å^3^), a volume of solvent-accessible voids of 476.1 Å^3^ (*ca.* 18%) and an electron count per unit cell of 192.4 were determined. Considering that one dichloromethane packing solvent molecule possessed 42 electrons, the calculated total electron count per unit cell (*Z* = 4, *Cc*) was 168 electrons, in agreement with the SQUEEZE calculated value. Each dichloromethane packing solvent involved the next three non-hydrogen atoms, summing up to 12 non-hydrogen atoms in the VOIDs. As it is reasonable to assume a volume per non-hydrogen atom of disordered solvents in VOIDS of 40 Å^3^, an overall VOID volume of 480 Å^3^ was calculated. This is in excellent agreement with the SQUEEZE calculated value of 476.1 Å^3^.

## 3. Results and discussion

### 3.1. Synthesis

**Scheme 1:**
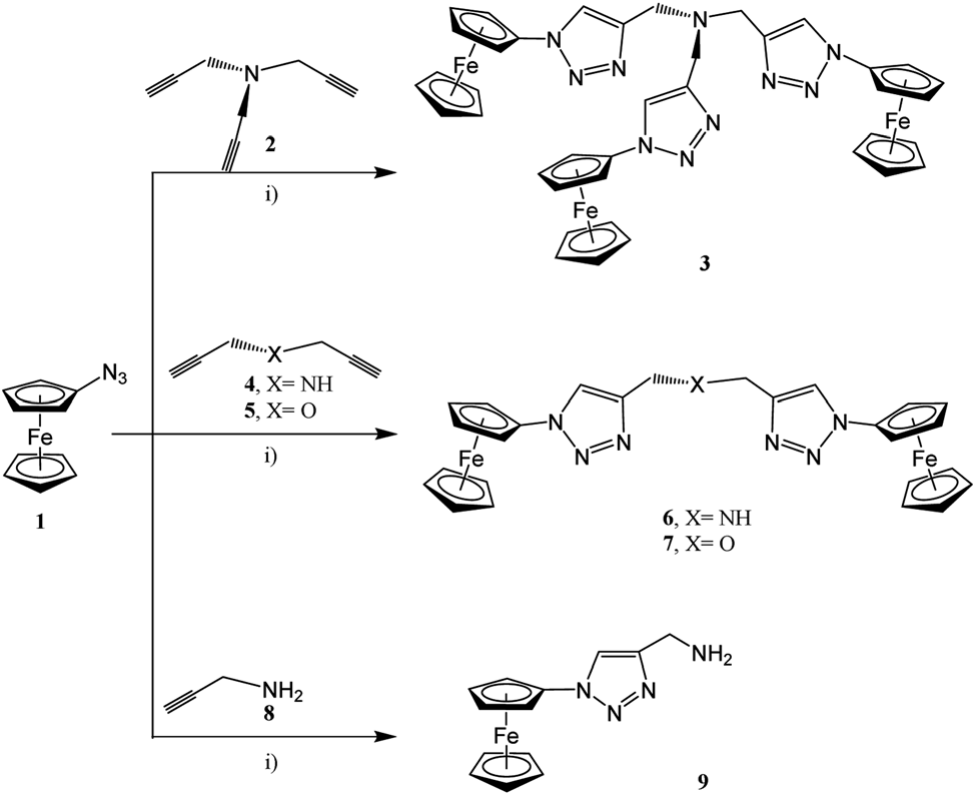
Reaction of 1 with 2, 4, 5, or 8 forming the ferrocenyl triazoles 3, 6, 7, and 9; (i) 1st step: solution A: thf–H_2_O (ratio 7 : 1, v/v), for 3: (3.3 equiv. of 1, 1.0 equiv. of 2), for 6 or 7: (2.2 equiv. of 1, 0.15 equiv. of 3, 1.0 equiv. of 4 or 5, respectively), for 9: (1.1 equiv. of 1, 0.15 equiv. of 3, 1.0 equiv. of 8); 2nd: 0.3 equiv. of CuSO_4_ in H_2_O; 3rd: 3.0 equiv. of Na–ascorbate in H_2_O; 4th: 25 °C, 48 h.; 5th: washing with NH_3_ (until colorless), then with H_2_O (pH = 7.00).^52,53,76–80^

In accordance with the classical click chemistry protocol, 1-azidoferrocene (1), which is accessible by the reaction of 1-bromoferrocene with sodium azide in the presence of copper(i) bromide,^52,74^ was reacted with 3.3 equiv. of tripropargylamine in a tetrahydrofuran–water mixture in the ratio of 6 : 1 (v/v) accompanied by the addition of an aqueous solutions of CuSO_4_ and Na–ascorbate at 25 °C for 48 h. After appropriate work-up, the ferrocenyl–triazole complex 3 was obtained in a yield 94% (Experimental section, Scheme 1).^75–81^ However, when using this classical click synthesis protocol for the preparation of 6 and 7 only very low yields could be obtained, while the synthesis of 9 was unsuccessful. Hence, complex 3 was used as an alternative stabilizing ligand instead of tris-(benzyltriazolylmethyl)amine (TBTA) for *in situ* generation of the respective Cu(i) catalyst.^78^ Thus, when 1 was reacted with 2.2 equiv. of dipropargylamine or dipropargyl ether and 1.1 equiv. of propargylamine, then 6, 7, and 9 could be isolated in 88–92% yields (Experimental section, Scheme 1).

### 3.2. Characterization

Compounds 3, 6, 7, and 9 were obtained as air-stable yellow-brownish solid materials at room temperature. They were characterized by IR, and NMR spectroscopy (^1^H, ^13^C{^1^H}), high-resolution ESI-TOF mass spectrometry, elemental analysis, and electrochemistry experiments. The molecular structures of 3, 7, and 9 in the solid state were determined by single-crystal X-ray diffraction analysis.

The presence of the amine functionality in 9 and 6 could be confirmed by IR spectroscopy by intense N–H stretching and bending vibrations at 3138 and 1206 cm^−1^ (9) or 3138 and 1106 cm^−1^ (6). New bands at 1107 cm^−1^ with a moderate intensity grew which were characteristic of ν_CN_ vibrations in 3, 6, and 7. In addition, stretching vibrations (1218 cm^−1^ (3), 1220 cm^−1^ (6), 1222 cm^−1^ (7), and 1213 cm^−1^ (9)) typical for aromatic amines confirmed the successful introduction of a triazole functionality. Within 7, the ether functionality was characterized by a C–O stretching vibration at 1042 cm^−1^.

In the ^1^H NMR spectra of 3, 6, 7, and 9, the Fc units showed the expected pattern between 4.2–4.9 ppm (Experimental). While the C_5_H_5_ protons resonated as a singlet at 4.2 ppm, and the C_5_H_4_ α and β protons appeared as pseudo-triplets at 4.3 and 4.8 ppm with *J*_HH_ = 2.0 Hz. The triazole-heterocyclic units showed singlets at 8.09 ppm (3), 7.87 ppm (6), 7.85 ppm (7), and 7.69 ppm (9). The CH_2_ protons could be detected as singlets at 3.87 ppm (3), 4.02 ppm (6), 4.27 ppm (7), and 4.04 ppm (9). The protons of the amine functionality in 6 and 9 were found as singlets at 2.04 and 1.57 ppm. Within the ^13^C NMR spectra, all the complexes showed individual signals for the triazole moieties at *ca.* 145 and 122 ppm, as well as for the ferrocenyl moieties between 62 and 94 ppm in the spectral region. Also, –CH_2_– signals were detected at different chemical shifts (47 (3), 44 (6), 62 (7), and 38 (9)), reflecting the electron-donating effect of the heteroatoms connected.^82–84^

**Fig. 1:**
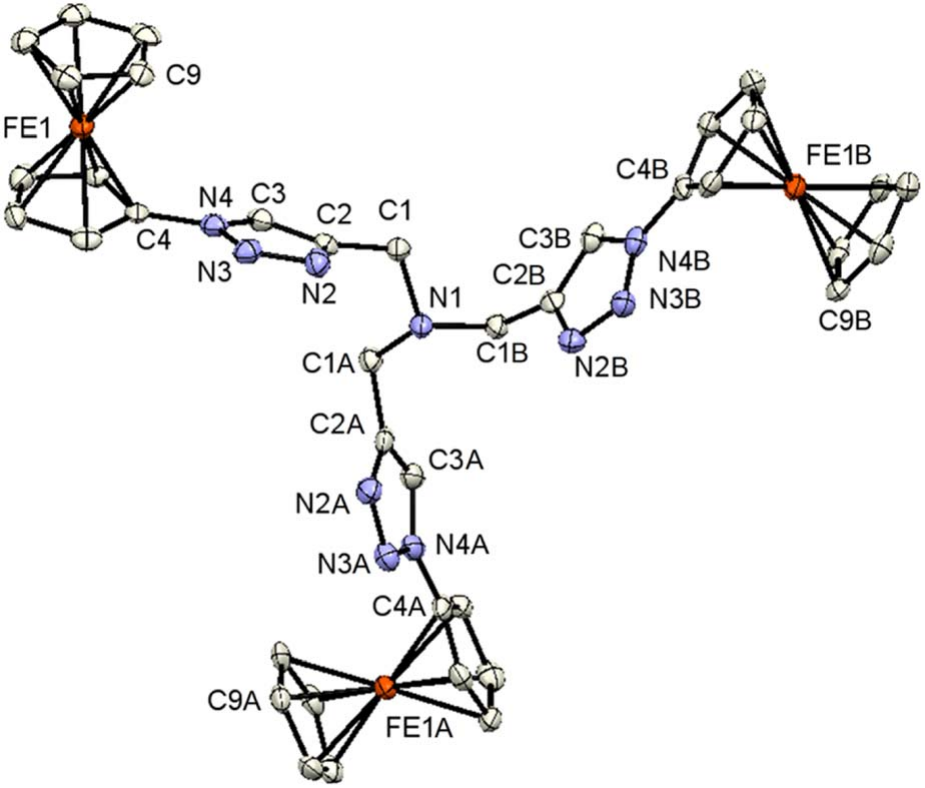
ORTEP (50% probability level) of the molecular structure of 3 with the atom numbering scheme. Hydrogen atoms are omitted for clarity. Symmetry operation for generating equivalent atoms: ‘A’ = −*y* + 1, *x* − *y*, *z*. ‘B’ = −*x* + *y* + 1, −*x* + 1, *z*.

**Fig. 2:**
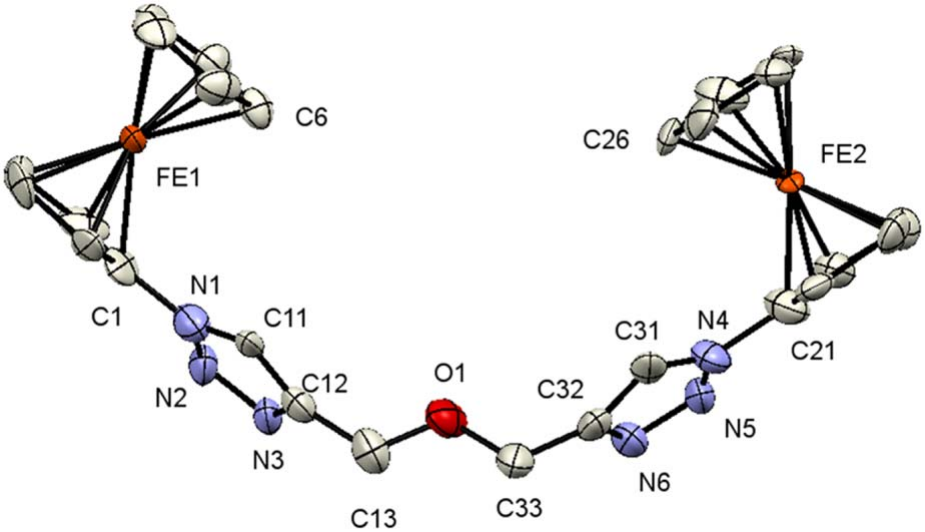
ORTEP (50% probability level) of the molecular structure of 7 with the atom numbering scheme. Hydrogen atoms are omitted for clarity.

**Fig. 3:**
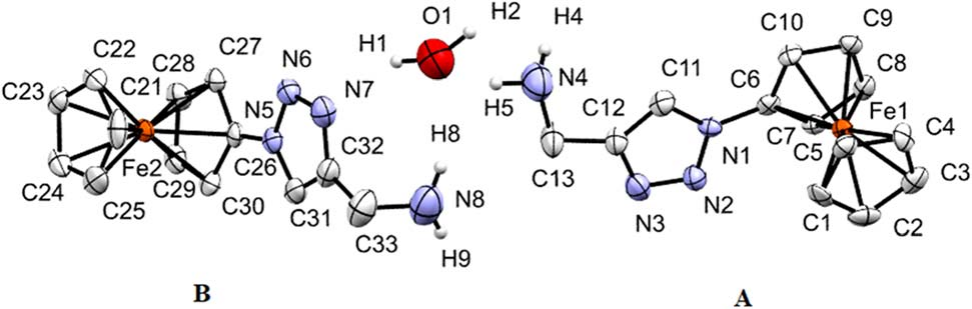
ORTEP (50% probability level) of the molecular structure of 9 with the atom numbering scheme. Hydrogen atoms are omitted for clarity. The two crystallographically different molecules of 9 are denoted as A (including Fe1) and B (including Fe2).

Single crystals of 3, 7, and 9 suitable for X-ray diffraction analysis were obtained by the diffusion of pentane into a dichloromethane solution containing either 3, 7, or 9 at ambient temperature. The crystal and structure refinement data are presented in Table 1. The molecular structures of 3, 7, and 9 with the atom labeling scheme are provided in Fig. 1–3. The bond distances (Å) and valence and torsion angles (deg) are given in Table S1 (see the ESI†).

**Table 1:** Crystal and structure refinement data for 3, 7, and 9

	3	7	9
Chemical formula	C_39_H_36_Fe_3_N_10_	C_26_H_22_Fe_2_N_6_O	C_26_H_30_Fe_2_N_8_O
Formula weight/g mol^−1^	812.33	546.19	582.28
Temperature/K	120	100	100
Wavelength/Å	0.71073	1.54178	1.54178
Crystal system	Trigonal	Monoclinic	Monoclinic
Space group	*R*3*c*	*Cc*	*C*2/*c*
*a*/Å	22.8746(12)	31.1767(14)	41.1986(19)
*b*/Å	22.8746(12)	5.6862(3)	5.6676(3)
*c*/Å	10.8056(9)	15.4318(7)	28.3277(15)
*β*/°	90°	104.342(2)^o^	131.772(2)^o^
*γ*/°	120°	90°	90°
*V*/Å^−3^	4896.5(7)	2650.4(2)	4933.1(4)
*ρ*_calcd_/g cm^−3^	1.653	1.369	1.568
*F*(000)	2508	1120	2416
Crystal size/mm	0.30 × 0.04 × 0.04	0.40 × 0.20 × 0.02	0.20 × 0.04 × 0.04
*Z*	6	4	8
Max. and min. transmission	1.00, 0.69801	0.81, 0.23	0.78, 0.48
*μ*/mm^−1^	1.365	9.009	9.738
*θ* range/°	3.085–29.013	2.926–65.989	2.876–63.683
Index ranges	−27 ≤ *h* ≤ 30	−36 ≤ *h* ≤ 35	−46 ≤ *h* ≤ 47
	−30 ≤ *k* ≤ 30	−6≤*k* ≤ 5	−6≤*k* ≤ 6
	−14 ≤ *l* ≤ 14	−18 ≤ *l* ≤ 17	−32 ≤ *l* ≤ 25
Total/unique reflections	11 602/2660	11 512/4101	20 136/4024
Data/restraints/parameters	2660/1/157	4101/32/317	4024/16/373
*R*_int_	0.0939	0.0573	0.1012
*R*_1_, w*R*_2_ [*I* ≥ *σ*(*I*)]	0.0486, 0.0788	0.07971, 0.2369	0.0879, 0.1640
*R*_1_, w*R*_2_ (all data)	0.1036, 0.0952	0.1076, 0.2480	0.1218, 0.1846
Goodness-of-fit *S* on *F*^2^	0.984	1.062	1.159
Largest diff. peak and hole/e·Å^−3^	0.537, −0.518	1.073, −0.664	0.617, −0.566
Absolute structure parameter^73^	0.01(2)	0.27(2)	—

Compound 3 crystallizes in the trigonal space group *R*3*c*:*H*. The molecular architecture in which three ferrocenyl-1*H*-1,2,3-triazole entities are bonded to a trimethylamine scaffold was unambiguously confirmed. The Fc groups adopt intermediate conformations between the staggered and the eclipsed form (C9–Fe1–C4–N4 = 21.2(5)°).^85^ The intramolecular through-space distance between the Fe atoms is 13.054(3) Å. The 1*H*-1,2,3-triazole ring exhibits a planar structure, and is slightly twisted in relation to the attached cyclopentadienyl groups, forming a dihedral angle of 15.7(3)°. The dihedral angle between methanamine and 1*H*-1,2,3-triazole is 76.1(2)°.

All the other structural parameters were unexceptional and compared well with those of related species.^86,87^ On the other hand, in the solid state, C–H⋯N interactions occur between the 1*H*-1,2,3-triazole rings, leading to the formation of chains along the *a*-axis, as illustrated in Fig. S1 (see the ESI†). Within the solid-state analysis of compound 3, there were parallel-displaced π⋯π interactions noted between two cyclopentadienyl rings, resulting in the formation of three-dimensional chains along the *c*-axis (Fig. S2, see the ESI†).

Compound 7 crystallizes in the monoclinic, space group *Cc*. The molecule features a central O atom connected on either side by methyl-1-ferrocenyl-1*H*-1,2,3-triazoles, which are syn-oriented (Fig. 2). The cyclopentadienyl rings at Fe1/Fe2 adopt an eclipsed conformation (torsion angles C6–Fe1–C1–N1/C26–Fe2–C21–N4 = 2.8(9)/−1.4(8)°). As anticipated, the 1*H*-1,2,3-triazole ring displays a planar structure, and it exhibits a slight twist concerning the attached cyclopentadienyl groups, resulting in dihedral angles of 24(1)°/22(1)°. It is noteworthy that the bond lengths of C11–C12 (1.42(3) Å) and C31–C32 (1.45(3) Å) as formal double bonds are elongated compared to localized C=C double bonds (1.34 Å). In addition, the N1–N2 bond (1.43(2) Å) is longer than the localized N–N single bonds (1.37 (3) Å), while the N4–N3 bond length is within the expected range for such compounds.^88,89^ The intermolecular separation between two cyclopentadienyl rings bonded to Fe2 is 3.31(3) Å, based on the bond separation between C21 and C26 and is almost identical compared to the situation around Fe1 with a separation of 3.32(2) Å between C1 and C6.^90,91^ All the remaining structural parameters are typical and align closely with those of related species.^85,86,92^ Interestingly, the C–H⋯π and C–H⋯ H–C interactions involving the Fc rings lead to the formation of a chain (Fig. S3 and S4, see the ESI†). Moreover, π⋯π interactions between cyclopentadienyl and the 1*H*-1,2,3-triazole ring were observed forming chains along the *b*-axis; graphical representations can be found in Fig. S3 (see the ESI†). Further interactions *via* C–H⋯N (Fig. S4, see the ESI†) lead to the formation of a 3D network.

Compound 9 crystallizes in the monoclinic space group *C*2/*c*. The structure of 9 is presented in Fig. 3, which shows that 9 crystallizes in the form of [(9)_2_ × H_2_O]. Thereby, the two crystallographically different molecules of 9 are denoted as A (including Fe1) and B (including Fe2) and within the asymmetric unit, one water molecule is present. In both molecules, the two cyclopentadienyl rings exhibit a tilt angle of 0.9(4)°/0.8(9)°, and they are approximately eclipsed with a twist angle of −1.1(8)° (N1–C6–Fe1–C1)/4.8(7)° (N5–C26–Fe2–C21) for the A and B molecules, respectively. The remaining geometrical parameters within the ferrocenyl framework are typical.^90^ As anticipated, the 1*H*-1,2,3-triazole ring is planar, and it exhibits a slight twist concerning the attached cyclopentadienyl groups, resulting in dihedral angles of 10.8(3)° and 6.8(8)° for A and B, respectively. The most significant difference between the two molecules comprising the asymmetric unit is evident in the dihedral angle between the methanamine and the 1*H*-1,2,3-triazole unit, measuring 7.3° for molecule A, in contrast to 73.1° for molecule B. Interestingly, the water molecule exists within the crystal packing connecting both molecules through hydrogen bonding between the NH_2_ groups and the H_2_O. Furthermore, a water molecule forms a hydrogen bond with the 1*H*-1,2,3-triazole ring of molecule B.

Through involving the Fc rings, C–H⋯π interactions between molecules A and B lead to the formation of a chain. Further interactions *via* C–H⋯π between Fc rings and 1,2,3-triazole rings lead to the formation of a 2D structure (Fig. S5 and S6, see the ESI†). In the perpendicular direction, further C–H⋯π interactions between Fc and 1*H*-1,2,3-triazole rings and C–H⋯π interactions between Fc rings beside C–H⋯N, CH_2_⋯C, N⋯N contacts and π⋯π stacking furnish the 3D structure (Fig. S7, see the ESI†).

### 3.3. Hirshfeld surfaces analysis

**Fig. 4:**
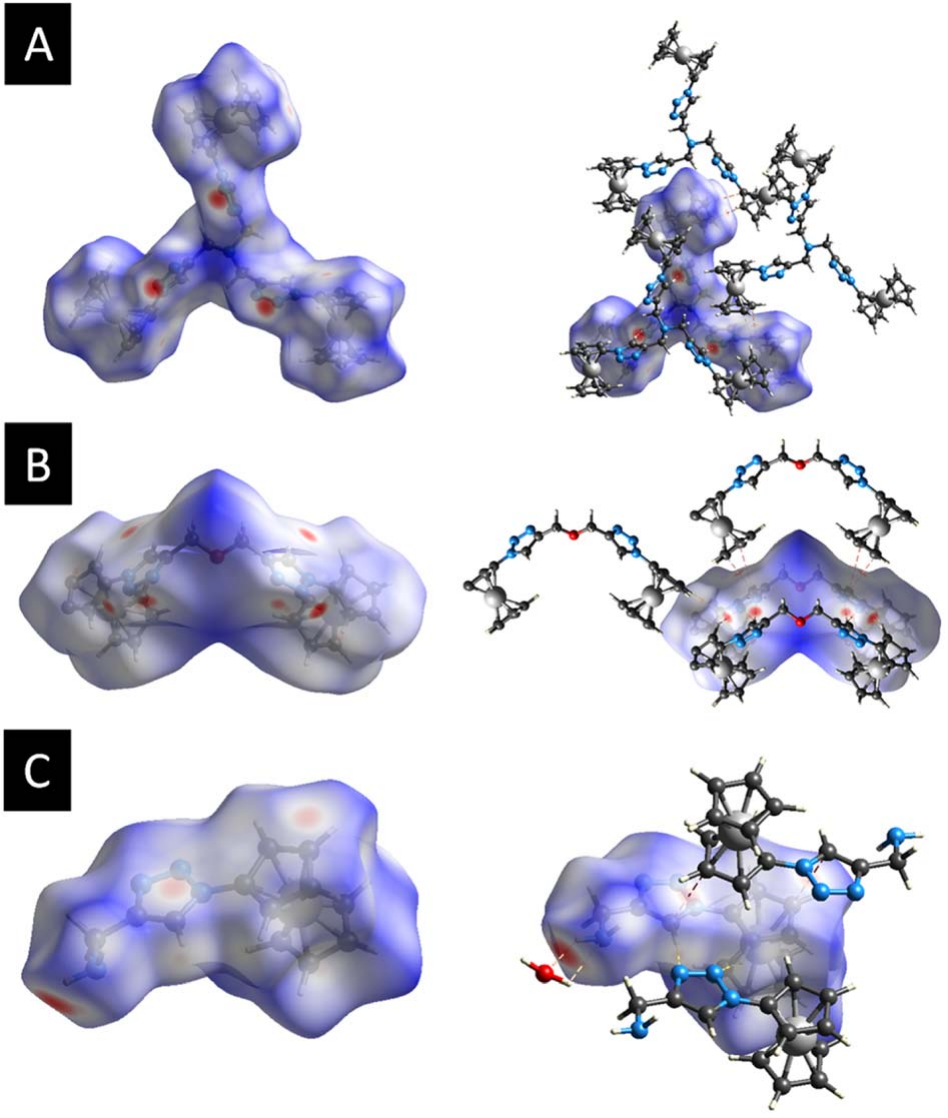
(A) Hirshfeld surfaces of 3 mapped over *d*_norm_, (B) Hirshfeld surfaces of 7 mapped over *d*_norm_, (C) Hirshfeld surfaces of 9 mapped over *d*_norm_.

Further analysis of the molecular packing scheme was performed by calculating the Hirshfeld surfaces and two-dimensional fingerprint plots (overall and decomposed) employing Crystal Explorer 17 (ref. 93) using standard protocols^94^ to discern and clarify the impact of the noteworthy intermolecular interactions observed in the crystal packing, which were consistent across the three compounds, prompting an examination of the role played by weak non-covalent interactions in the supramolecular assembly. This included exploring the significance of H⋯N, H⋯O, and π⋯π stacking interactions in establishing the configuration of the extended structure.^95–100^Fig. 4 shows the Hirshfeld surfaces mapped over the *d*_norm_ of 3, 7, and 9. The 2D fingerprint plots for 3, 7, and 9 are shown in Fig. S8–S10 (see the ESI†).

For each compound, in the crystal packing the bright and deep-red spot in the Hirshfeld surface indicates the closest distance between the atoms at the exterior (*d*_e_) and interior (*d*_i_) of the compound. Fingerprint plots (Fig. S8–S10, refer to the ESI†) illustrate that H⋯H interactions predominantly govern the surface contacts, which aligns with expectations given the abundance of H atoms in the molecule. Additionally, H⋯C/C⋯H and H⋯N/N⋯H contacts also play notable roles in the surface interactions (Table S2, see the ESI†).

Fig. 4a displays the Hirshfeld surfaces of 3. The prominent red areas on the Hirshfeld surfaces represent classical H⋯N/N⋯H hydrogen bonds, while the less pronounced red circles denote weaker π⋯π stacking contacts. In the *d*_norm_ map of 7, the dark red regions are associated with N⋯H/N⋯H hydrogen bonds and π⋯π stacking interactions, while the bright-red regions are attributed to C–H⋯π interactions (Fig. 4b). Hydrogen bonding interaction between 9 and the co-crystallized water molecule in the crystal structure was observed in the Hirshfeld surfaces. Fig. 4c represents the Hirshfeld surface of 9. As expected, deep-red spots were observed corresponding to the presence of hydrogen bonds (H⋯O/O⋯H, H⋯N/N⋯H), while the bright-red spots corresponded to the presence of C–H⋯π stacking.

### 3.4. Electrochemistry

**Fig. 5:**
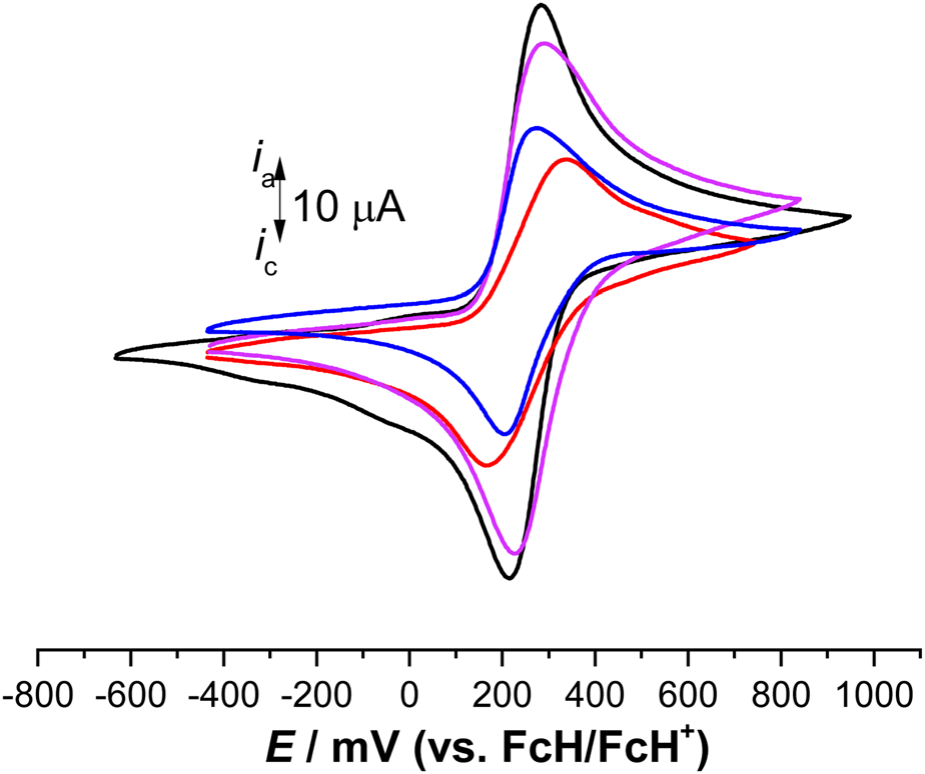
Cyclic voltammograms of 1.0 mM solution of 3 (red line), 6 (violet line), 7 (black line), and 9 (blue line), scan rate 100 mV s^−1^ in anhydrous dichloromethane with [N^*n*^Bu_4_][PF_6_] (0.1 mol L^−1^) used as a supporting electrolyte at 25 °C.^33^

The electrochemical behavior of 3, 6, 7, and 9 was investigated by cyclic voltammetry (CV) using an anhydrous dichloromethane solution of [N^*n*^Bu_4_][B(C_6_F_5_)_4_] (0.1 mol L^−1^) as a supporting electrolyte.^32,33,38,54–56,59,101^ The electrochemical measurements were conducted at 25 °C under an argon atmosphere and referenced against the potential of the FcH/FcH^+^ redox couple. In all compounds, reversible and well-defined redox events were observed, as illustrated in Fig. 5. The electrochemical data are consolidated in Table 1. The multicyclic experiments indicated the stability of the Fc/Fc^+^ redox couples for all the compounds.

In the cyclic voltammetry (CV) data, concerning the redox event of ferrocene (FcH/FcH^+^), the formal potentials were shifted toward anodic potentials (*E*°′ = 251 mV (3), 257 mV (6), 249 mV (7), 239 mV (9)), due to the electron-withdrawing character of the triazole moieties (Fig. 5, Table 2).^102^ The large Δ*E*_p_ value of 176 mV for 3 suggests that three individual reversible one-electron processes exist in a close potential range, indicating a certain degree of electrostatic interaction between the Fc/Fc^+^ moieties.^33,103–106^ The cyclic voltammetry (CV) data for compounds 6 and 7 revealed the presence of two distinct reversible one-electron processes superimposed on each other (Δ*E*_p_ = 66 mV for 6, 65 mV for 7). This suggests the absence of electronic and electrostatic interactions between Fc/Fc^+^, as illustrated in Fig. 5 and summarized in Table 2. The current for 6 and 7 was twice as intense as that of 9 (Fig. 5). Hence, the concurrent oxidation of the Fc units in 6 and 7 resulted in a lower thermodynamic stability of the mono-oxidized [6]^+^ and [7]^+^ species compared to the isovalent species [6]^2+^ and [7]^2+^, respectively. This implies a negligible electronic interaction between the Fc/Fc^+^ units.^105,107^

**Table 2:** Cyclic voltammetry data for 3, 6, 7, and 9 in dichloromethane using [N^*n*^Bu_4_][B(C_6_F_5_)_4_] (0.1 mol L^−1^) as a supporting electrolyte at 25 °C

Compd	*E*°′^a^ (mV)	Δ*E*_p_^b^ (mV)	*i*_pc_/*i*_pa_
3	251	176	1.01
6	257	66	1.14
7	249	65	1.09
9	239	62	0.96

a *E*°′ = formal potential.

b Δ*E*_p_ = difference between the oxidation and reduction peak potentials.

It is noteworthy that the cationic species [3]^*n*+^ (*n* = 1, 2, or 3), [6]^*n*+^ (*n* = 1 or 2), [7]^*n*+^ (*n* = 1 or 2), and [9]^+^ did not precipitate during the oxidation, neither at the electrode surface nor in the electrochemical cell. Therefore, even the fully oxidized cations remained soluble under the measurement conditions employed. However, *in situ* spectroelectrochemical-NIR measurements were not conducted for 3 due to the negligible redox separations, the connectivity pattern of the molecules, and reports from previous investigations on compounds containing sp^3^-hybridized bridging atoms.^104,108,109^

### 3.5. Electrochemical sensing

#### 3.5.1. Electrochemical characterization of the modified electrodes (3, 6, 7, or 9@SPCE)

**Fig. 6:**
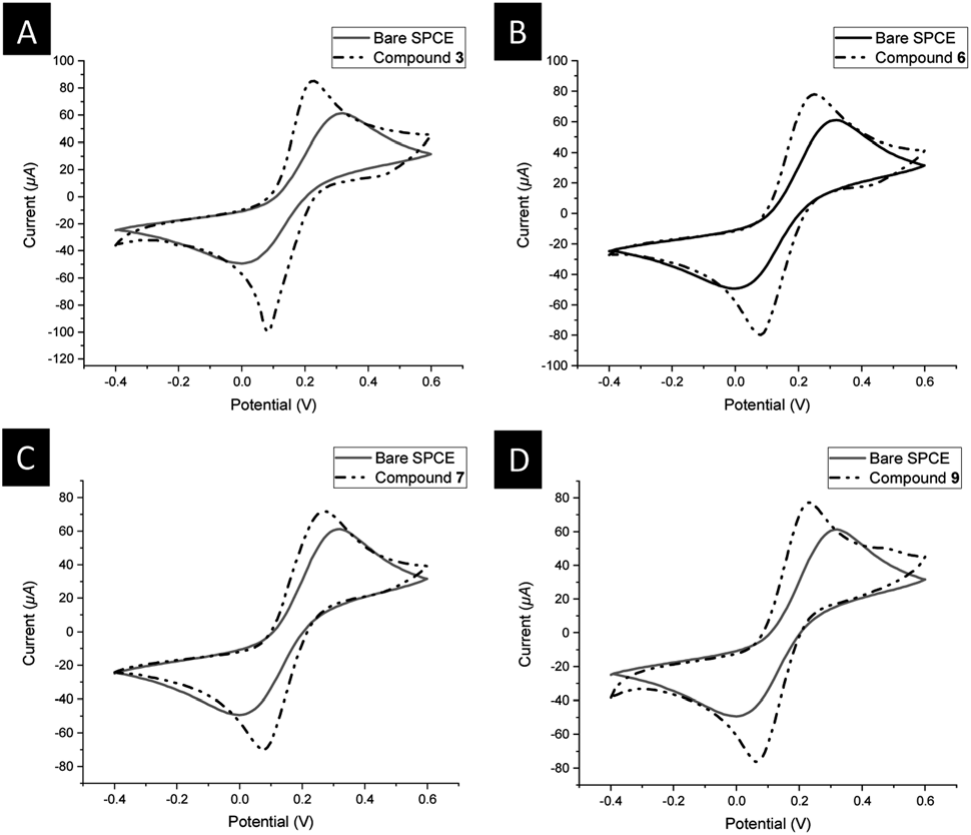
Cyclic voltammograms of 5.0 mM [Fe(CN)_6_]^3−/4−^ using: (A) bare SPCE (solid line), 3@SPCE (dashed dotted line); (B) bare SPCE (solid line), 6@SPCE (dashed dotted line); (C) bare SPCE (solid line), 7@SPCE (dashed dotted line); (D) bare SPCE (solid line), 9@SPCE (dashed dotted line); scan rate 100 mV s^−1^ in aqueous solution containing 0.1 M KCl used as a supporting electrolyte at 25 °C.

Measurements of the modified electrodes were performed using cyclic voltammetry techniques for an aqueous solution of 5.0 mM [Fe(CN)_6_]^3−/4−^ containing 0.1 M KCl. In the cyclic voltammetry (CV) measurements of the unmodified screen-printed carbon electrode (SPCE), a pair of weak redox peaks with a peak-to-peak separation (Δ*E*_p_ = 320 mV) was observed (Fig. 6, solid line CV), attributed to the slow electron-transfer rate at the interface. However, upon modification with the ferrocenyl–triazole complexes 3, 6, 7, or 9, it was evident that the current intensity of the redox peaks of [Fe(CN)_6_]^3−/4−^ increased, and the peak-to-peak separation (Δ*E*_p_ = 130 mV for 3, 170 mV for 6, 190 mV for 7, 170 mV for 9) decreased compared to the bare SPCE (Fig. 6). This behavior suggests that the presence of the ferrocenyl–triazole complexes 3, 6, 7, or 9 on the working electrode surface enhanced the effective surface area, thereby accelerating the electron transfer between the surface and the ferri/ferrocyanide solution. These assumptions deal with increasing the electrochemical effective surface area (ESA).^110,111^ In this regard, and following Randles–Ševčík's equation,^110,111^ the estimated electrochemical effective surface areas (0.0994 cm^2^ (for 3), 0.0992 cm^2^ (for 6), 0.0944 cm^2^ (for 7), and 0.0989 cm^2^ (for 9)) were found to be larger than that for the SPCE (0.0761 cm^2^). It was noticeable that 3@SPCE showed the highest ESA compared to the others, which was in agreement with the electron-transfer processes as well as the peak-to-peak separation value. Such behavior could be attributed to the presence of a higher content of ferrocenyl and/or triazole functionality among the other ferrocenyl–triazole complexes (Scheme 1 and Fig. 6).

#### 3.5.2. Electrochemical detection of Cd^2+^, Pb^2+^, Cu^2+^, Mn^2+^, Co^2+^, Ni^2+^, and Zn^2+^ using the modified electrodes (3, 6, 7, or 9@SPCE)

**Fig. 7:**
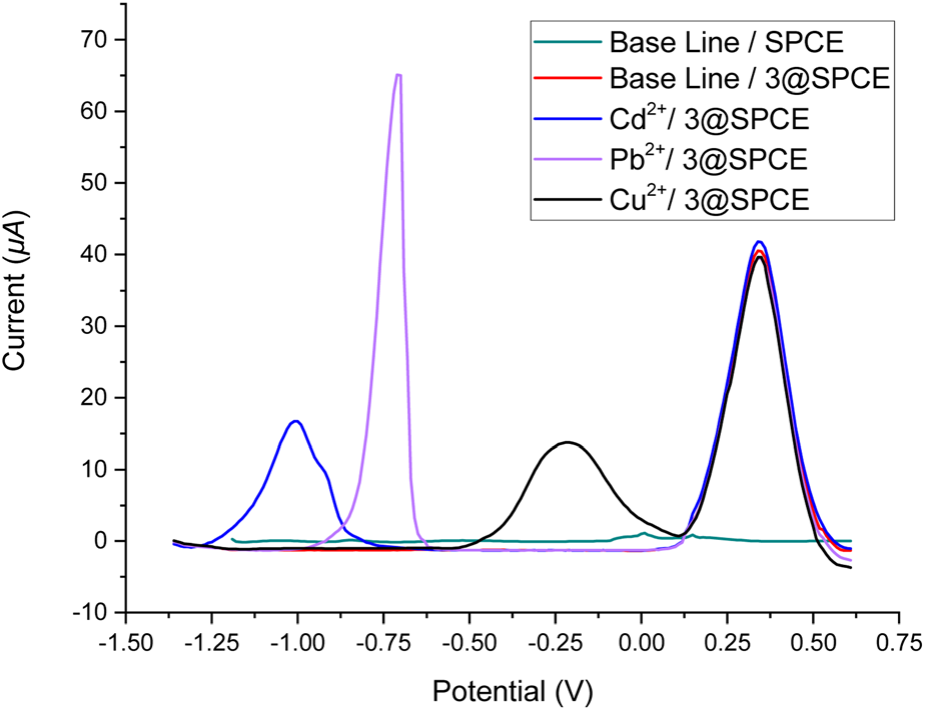
Square wave voltammograms of ABS solutions of Cd^2+^, Pb^2+^, and Cu^2+^ (250 μM) using a drop-casting method for the bare SPCE and 3@SPCE modified electrodes.

**Fig. 8:**
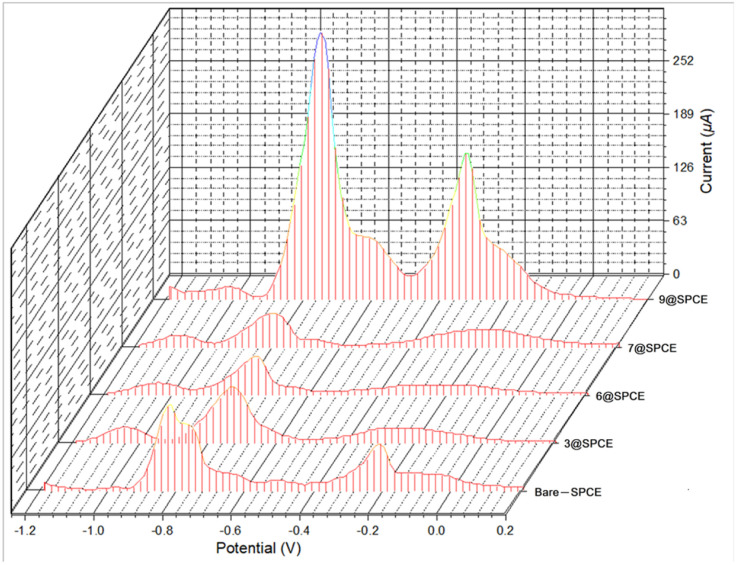
Square wave voltammograms for simultaneous detection of SAB solutions of Cd^2+^, Pb^2+^, and Cu^2+^ metal ions (150.0 μM) using a drop-casting method for bare SPCE and the modified electrodes 3@SPCE, 6@SPCE, 7@SPCE, and 9@SPCE.

Square wave voltammetry (SWV) of the modified electrodes (3, 6, 7, or 9@SPCE) of HAcO–NaAcO buffer solution (ABS (0.1 M), pH = 5.6) solutions containing Cd^2+^, Pb^2+^, Cu^2+^, Mn^2+^, Co^2+^, Ni^2+^, and Zn^2+^ (250.0 μM) individually were measured. In particular, the modified electrodes showed sensing responses toward the presence of Cu^2+^, Pb^2+^, and Cd^2+^ cations but displayed no affinity toward the other cations (Mn^2+^, Co^2+^, Ni^2+^, and Zn^2+^). As shown in Fig. 7 (data for 3 as representative), a characteristic redox event for the ferrocenyl–triazole functionalities was observed at 349 mV.^4,5,89^ The SWV measurements using modified electrodes showed three events at 1020, −730, and −180 mV *vs.* Ag/AgCl for Cd^2+^, Pb^2+^, and Cu^2+^, respectively. In addition, comparing the simultaneous detection behavior of the bare-electrode (SPCE) and the modified-SPCEs (Fig. 8) revealed that the modified electrodes displayed more reliable simultaneous detection for the addressed cations. It is noteworthy that the modified electrodes could potentially improve the simultaneous detection of Cd^2+^, Pb^2+^, and Cu^2+^, especially in the case of Cd^2+^ detection at −1020 mV, which was not displayed for the bare-electrode (SPCE) (Fig. 8, bare SPCE). These obvious findings validate the efficacy of the ferrocenyl–triazole complexes as electrode modifier materials for the detection of the specified analytes.

**Fig. 9:**
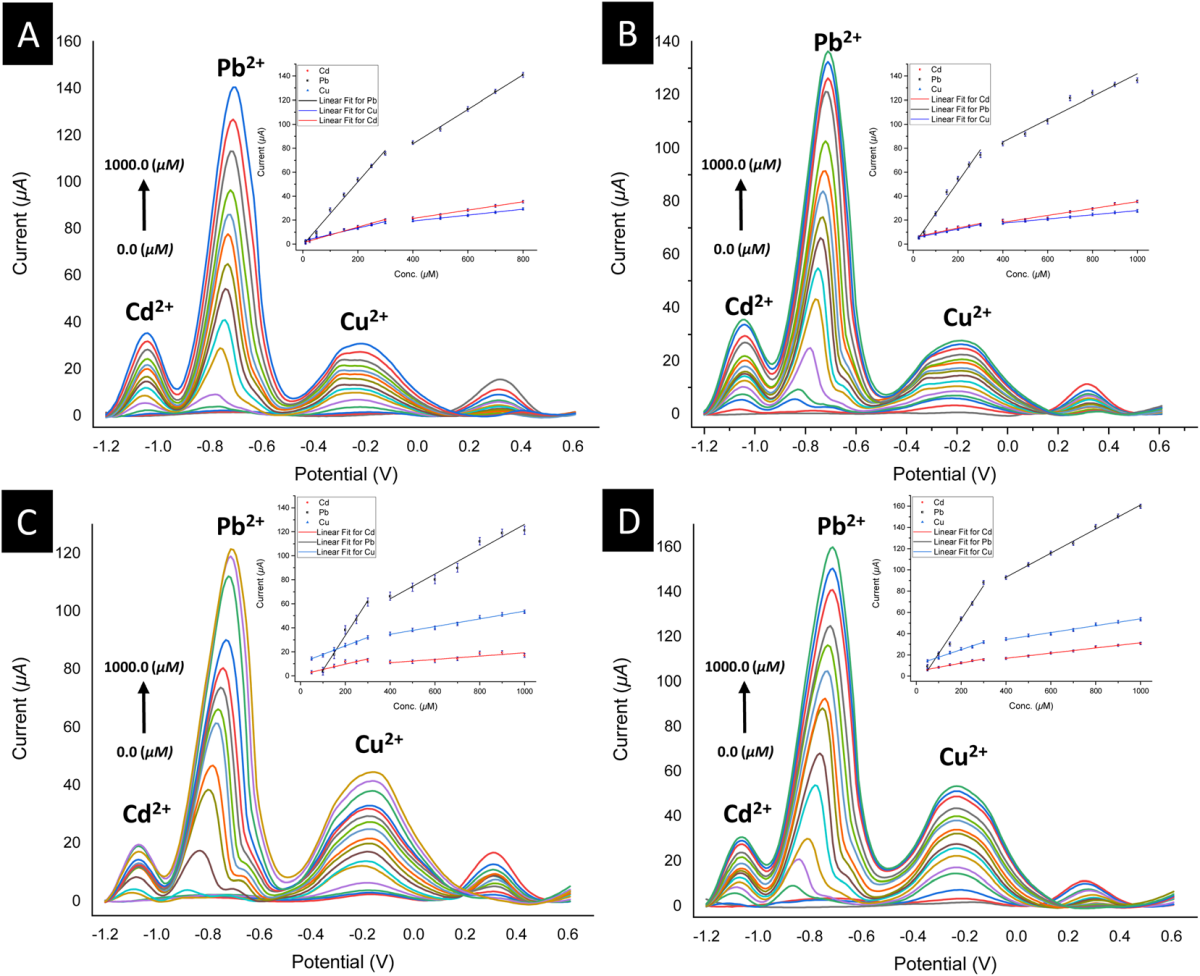
Square wave voltammograms (insets: linear calibration plots) for the simultaneous detection of SAB solutions of Cd^2+^, Pb^2+^, and Cu^2+^ metal ions (0.0–1000.0 μM) using a drop-casting method for the modified electrodes: (A) for 3@SPCE; (B) for 6@SPCE; (C) for 7@SPCE; (D) for 9@SPCE.

To validate the potential of the ferrocenyl–triazole materials more comprehensively for detecting multiple heavy metal ions simultaneously, we recorded their SWV responses with different concentrations (0–1000 μM) of Cd^2+^, Pb^2+^, and Cu^2+^ (Fig. 9, Table 3). It was noteworthy that the observed non-linearity and splitting in the calibration curve (Fig. 9) could be attributed to the interaction between heavy metal ions and the electrode surface. As the concentration of heavy metal ions increased, they adsorbed onto the electrode surface and formed a layer that inhibits electron transfer between the electrode and the solution.^112^ Nevertheless, the response patterns for the simultaneous detection of Cd^2+^, Pb^2+^, and Cu^2+^ could be easily discriminated with their response patterns and observed at the same detection potentials that appear as observed in Fig. 7, indicating that the potential separation was large enough to distinguish between the peaks. The limit of detections (LODs) of theses electrochemical sensors estimated based on a signal-to-noise ratio are summarized in Table 3 and were found to be comparable with electrochemical platforms for the detection of Cd^2+^, Pb^2+^, and Cu^2+^ in aqueous solutions (Table S3†).

**Table 3:** Linear regression equation, corresponding to the linear range, linear correlation coefficient, and LoD of the present sensing platform for Cd^2+^, Pb^2+^, and Cu^2+^ detection using the modified electrodes 3, 6, 7, or 9@SPCE

Electrode	[Cd^2+^]		[Pb^2+^]		[Cu^2+^]	
	Corresponding linear range		Corresponding linear range		Corresponding linear range	
	Linear regression equation		Linear regression equation		Linear regression equation	
	(*R*^2^)		(*R*^2^)		(*R*^2^)	
	LoD		LoD		LoD	
3@SPCE	(10–400) μM	(500–800) μM	(10–400) μM	(500–800) μM	(10–400) μM	(500–800) μM
	*y* = 1.11814 + 0.06508 × *x*	*y* = 7.48275 + 0.03476 × *x*	*y* = −0.9587 + 0.26426 × *x*	*y* = 26.3971 + 0.14327 × *x*	*y* = 2.58801 + 0.05381 × *x*	*y* = 9.3273 + 0.02473 × *x*
	(*R*^2^ = 0.98612)	(*R*^2^ = 0.99542)	(*R*^2^ = 0.99357)	(*R*^2^ = 0.99715)	(*R*^2^ = 0.98285)	(*R*^2^ = 0.99722)
	9.2 nM	—	6.3 nM	—	20.1 nM	—
6@SPCE	(50–400) μM	(500–1000) μM	(50–400) μM	(500–1000) μM	(50–400) μM	(500–1000) μM
	*y* = 5.65554 + 0.0387 × *x*	*y* = 6.67621 + 0.02873 × *x*	*y* = −1.23602 + 0.2667 × *x*	*y* = 47.46199 + 0.0944 × *x*	*y* = 4.92772 + 0.03808 × *x*	*y* = 10.44341 + 0.01749 × *x*
	(*R*^2^ = 0.93922)	(*R*^2^ = 0.95304)	(*R*^2^ = 0.98957)	(*R*^2^ = 0.95814)	(*R*^2^ = 0.99896)	(*R*^2^ = 0.99634)
	30.5 nM	—	8.7 nM	—	23.9 nM	—
7@SPCE	(50–400) μM	(500–1000) μM	(100–400) μM	(500–1000) μM	(50–400) μM	(500–1000) μM
	*y* = 0.86345 + 0.04393 × *x*	*y* = 5.34728 + 0.0138 × *x*	*y* = −24.24902 + 0.28923 × *x*	*y* = 22.75788 + 0.10343 × *x*	*y* = 10.73873 + 0.07073 × *x*	*y* = 21.68721 + 0.03217 × *x*
	(*R*^2^ = 0.91739)	(*R*^2^ = 0.77043)	(*R*^2^ = 0.98846)	(*R*^2^ = 0.95759)	(*R*^2^ = 0.9892)	(*R*^2^ = 0.98431)
	4.3 nM	—	29.0 nM	—	7.1 nM	—
9@SPCE	(50–400) μM	(500–1000) μM	(50–400) μM	(500–1000) μM	(50–400) μM	(500–1000) μM
	*y* = 4.29173 + 0.03969 × *x*	*y* = 7.02349 + 0.02437 × *x*	*y* = −10.79841 + 0.31988 × *x*	*y* = 47.6892 + 0.11351 × *x*	*y* = 10.75638 + 0.07072 × *x*	*y* = 21.54613 + 0.03241 × *x*
	(*R*^2^ = 0.98255)	(*R*^2^ = 0.98967)	(*R*^2^ = 0.98368)	(*R*^2^ = 0.99651)	(*R*^2^ = 0.9887)	(*R*^2^ = 0.98475)
	3.7 nM	—	32.0 nM	—	7.1 nM	—

## 4. Conclusions

In conclusion, the ferrocenyl–triazole compounds 3, 6, 7, and 9 were prepared starting from the reaction of 1-azidoferrocene (1) with propargylamine/propargyl ether in tetrahydrofuran–water mixtures accompanied by the addition of aqueous solutions of CuSO_4_ and Na–ascorbate. All the compounds were characterized by IR and NMR spectroscopy (^1^H, ^13^C{^1^H}), high-resolution ESI-TOF mass spectrometry, as well as elemental analysis. The solid-state structures for 3, 7, and 9 were determined by single-crystal X-ray diffraction. The molecular structure of 3 involves a trimethylamine scaffold bonded to three ferrocenyl-1*H*-1,2,3-triazole entities, while compound 9 comprises ferrocenyl-1*H*-1,2,3-triazole entities bonded to a methylamine scaffold. On the other hand, the molecular structure of 7 consists of a central oxygen (O) atom linked on both sides to methyl-1-ferrocenyl-1*H*-1,2,3-triazoles. Several intermolecular interactions such as π⋯π and C–H⋯N, between the cyclopentadienyl and triazole ligands lead to the formation of chains and 3D network structures within compounds 3, 7, and 9. Hirshfeld surface analysis confirmed the presence of similar intermolecular contact patterns. Within the electrochemical measurements (CV), all the compounds showed anodically shifted (relative to FcH/FcH^+^) reversible events, and the multicyclic measurements showed stable redox behaviors for the cationic species of [3]^*n*+^ (*n* = 1, 2 or 3), [6]^*n*+^ (*n* = 1 or 2), [7]^*n*+^ (*n* = 1 or 2), and [9]^+^. In the cyclic voltammetry (CV) curves of 6 and 7, two distinct reversible one-electron processes were superimposed, indicating the absence of electronic and electrostatic interactions between Fc/Fc^+^. In addition, the potential use of these compounds to fabricate 3, 6, 7, and 9@SPCE as electrochemical sensors for the detection of some heavy metals (Cu^2+^, Pb^2+^, and Cd^2+^) was successfully realized. These results open an avenue to the use of such ferrocene-triazole-containing materials in combination with carbon-based nanomaterials and/or conductive polymers as promising modifiers with compatible sensing properties toward heavy metals for real applications.^5,87^

## Author contributions

Khaled Al Khalyfeh: conceptualization, project administration, investigation, formal analysis, methodology, validation and writing – review & editing. Asma Ghazzy: conceptualization and formal analysis. Randa Al-As'ad: investigation. Tobias Rüffer: formal analysis. Olfa Kanoun: resources and writing – review & editing. Heinrich Lang: resources and writing – review & editing.

## Conflicts of interest

The authors declare no conflict of interest.

## Supplementary Material

RA-014-D4RA04023F-s001

RA-014-D4RA04023F-s002
