# Supplementary material for: Ferrocenyl–triazole complexes and their use in heavy metal cation sensing
Source: RSC Adv. 2024 Jun 28;14(29):20572–84. doi: 10.1039/d4ra04023f (PMC11211737; doi:10.1039/d4ra04023f)
Supplement: RA-014-D4RA04023F-s001 [file RA-014-D4RA04023F-s001.pdf]

Electronic Supplementary Material (ESI) for RSC Advances.
This journal is © The Royal Society of Chemistry 2024

For Supporting Information

Ferrocenyl-Triazole Complexes and their Use in Heavy Metal Cation Sensing

Khaled Al Khalyfeh[a*], Asma Ghazzy[b,c], Randa M Al-As'ad[a], Tobias Ruffer[d], Olfa Kanoun[e], Heinrich Lang[f]

a. Department of Chemistry, Faculty of Natural Science, Al-Hussein Bin Talal University, Ma'an 71111, Jordan.
b. Faculty of Pharmacy. Faculty of Pharmacy and Applied Medical Sciences, Al-Ahliyya Amman University, Amman 19328, Jordan.
c. Pharmacological and Diagnostic Research Center, Faculty of Pharmacy and Allied Medical Sciences, Al-Ahliyya Amman University. Amman 19328, Jordan.
d. Department of Inorganic Chemistry, Chemnitz University of Technology, 09126 Chemnitz, Germany..
e. Professorship Measurement and Sensor Technology, Chemnitz University of Technology, 09126 Chemnitz, Germany.
f. Research Center for Materials, Architectures and Integration of Nanomembranes (MAIN), Research Group Organometallics, Chemnitz University of Technology, 09126 Chemnitz, Germany.

* E-mail: k.khalyfeh@ahu.edu.jo.

Table of Content:

Table S 1. Selected bond distances (Å), angles (°), and plane intersections (°) of **3**, **7** and **9**........3

Table S 2. Percentage contributions to the Hirshfeld surface of **3**, **7** and **9**. 4

Table S 3. Comaprison of LODs for different electrochemical sensor for Cd^{2+}, Pb^{2+} and Cu^{2+} determination...4

Figure S 1. Illustration of C–H···N (black) contacts in **3**. 4

Figure S 2. Illustration of $\pi ... \pi$ stacking in **3**. 5

Figure S 3. Illustration of C–H···π contacts, C–H ··· H–C stacking and $\pi ... \pi$ stacking in **7**. 5

Figure S 4. Illustration of C–H···π and C–H···N contacts in **7**. 6

Figure S 5. Intermolecular interaction within the crystal backing of **9**. 6

Figure S 6. Intermolecular interaction within the crystal backing of **9**. 6

Figure S 7. Illustration of $\pi ... \pi$ stacking, C–H···O contacts, C–H···C contacts, C–H···C contacts, N ··· N contacts and CH_2···C contacts in 9...7

Figure S 8. Full 2-D fingerprint plot for **3** representing intermolecular interactions. 7

Figure S 9. Full 2-D fingerprint plot for **7** representing intermolecular interactions. 8

Figure S 10. Full 2-D fingerprint plot for **9** representing intermolecular interactions. 8

Figure S 11. ^{1}H NMR spectrum of **3** in $CDCl_3$. 9

Figure S 12. ^{1}H NMR spectrum of **6** in $CDCl_3$. 9

Figure S 13. ^{1}H NMR spectrum of **7** in $CDCl_3$. 10

Figure S 14. ^{1}H NMR spectrum of **9** in $CDCl_3$. 10

Figure S 15. ^{13}C NMR spectrum of **3** in $CDCl_3$. 11

Figure S 16. ^{13}C NMR spectrum of **6** in $CDCl_3$. 11

Figure S 17. ^{13}C NMR spectrum of **7** in $CDCl_3$. 12

Figure S 18. ^{13}C NMR spectrum of **9** in $CDCl_3$. 12

Figure S 19. ESI-TOF Mass spectrum of **3**. 13

Figure S 20. ESI-TOF Mass spectrum of **6**. 13

Figure S 21. ESI-TOF Mass spectrum of **7**. 14

Figure S 22. ESI-TOF Mass spectrum of **9**. 14

Figure S 23. IR spectrum of **3**. 15

Figure S 24. IR spectrum of **6**. 15

Figure S 25. IR spectrum of **7**. 16

Figure S 26. IR spectrum of **9**. 16

Table S 1. Selected bond distances (Å), angles (°), and plane intersections (°) of **3**, **7** and **9**.

Selected bond distances (Å)					
**3**		**7**		**9**	
C(1)–C(2)	1.497(8)	C(1)–N(1)	1.45(2)	C(11)–C(12)	1.364(11)
C(1)–N(1)	1.477(6)	C(11)–C(12)	1.42(3)	C(11)–N(1)	1.352(9)
C(2)–C(3)	1.371(9)	C(11)–N(1)	1.38(3)	C(12)–C(13)	1.504(11)
C(2)–N(2)	1.358(8)	C(12)–C(13)	1.48(3)	C(12)–N(3)	1.378(11)
C(3)–N(4)	1.346(8)	C(12)–N(3)	1.44(3)	C(13)–N(4)	1.513(14)
C(4)–N(4)	1.427(8)	C(13)–O(1)	1.41(2)	C(31)–C(32)	1.361(12)
N(2)–N(3)	1.330(7)	C(21)–N(4)	1.48(2)	C(31)–N(5)	1.346(10)
N(3)–N(4)	1.343(7)	C(31)–C(32)	1.45(3)	C(32)–C(33)	1.492(12)
		C(31)–N(4)	1.37(2)	C(32)–N(7)	1.372(12)
		C(32–C(33)	1.46(3)	C(33)–N(8)	1.440(13)
		C(32)–N(6)	1.41(3)	C(6)–N(1)	1.410(9)
		C(33)–O(1)	1.38(2)	N(1)–N(2)	1.350(9)
		N(1)–N(2)	1.43(2)	N(2)–N(3)	1.315(9)
		N(2)–N(3)	1.33(2)	N(5)–N(6)	1.351(9)
		N(4)–N(5)	1.37(3)	N(6)–N(7)	1.330(9)
		N(5)–N(6)	1.32(2)		
Selected bond angles (°)					
**3**		**7**		**9**	
C(1)#1-N(1)-C(1)	109.5(4)	C(11)-C(12)-C(13)	125(2)	C(11)-C(12)-C(13)	130.6(8)
C(1)#1-N(1)-C(1)#2	109.5(4)	C(11)-C(12)-N(3)	111.0(17)	C(11)-C(12)-N(3)	108.1(7)
C(1)#2-N(1)-C(1)	109.5(4)	C(11)-N(1)-C(1)	127.5(18)	C(11)-N(1)-C(6)	127.9(7)
C(3)-C(2)-C(1)	128.0(7)	C(11)-N(1)-N(2)	114.3(16)	C(12)-C(11)-N(1)	105.0(7)
C(3)-N(4)-C(4)	127.6(6)	C(31)-C(32)-C(33)	126(2)	C(31)-C(32)-C(33)	129.6(9)
N(1)-C(1)-C(2)	112.5(5)	C(31)-N(4)-C(21)	123.5(18)	C(31)-C(32)-N(7)	108.6(8)
N(2)-C(2)-C(1)	123.7(6)	C(31)-N(4)-N(5)	116.3(16)	C(31)-N(5)-C(26)	127.8(7)
N(2)-C(2)-C(3)	108.3(6)	C(33)-O(1)-C(13)	112.3(16)	N(2)-N(1)-C(11)	111.0(6)
N(2)-N(3)-N(4)	106.9(5)	N(1)-C(11)-C(12)	100.9(19)	N(2)-N(1)-C(6)	121.1(6)
N(3)-N(2)-C(2)	108.7(5)	N(2)-N(1)-C(1)	118.1(16)	N(2)-N(3)-C(12)	108.8(7)
N(3)-N(4)-C(3)	111.0(5)	N(2)-N(3)-C(12)	108.1(17)	N(3)-C(12)-C(13)	121.2(8)
N(3)-N(4)-C(4)	121.1(6)	N(3)-C(12)-C(13)	124.5(19)	N(3)-N(2)-N(1)	107.1(6)
N(4)-C(3)-C(2)	105.0(6)	N(3)-N(2)-N(1)	105.6(15)	N(4)-C(13)-C(12)	112.0(8)
		N(4)-C(31)-C(32)	99.4(18)	N(5)-C(31)-C(32)	104.6(8)
		N(5)-N(4)-C(21)	120.2(17)	N(6)-N(5)-C(26)	120.0(6)
		N(5)-N(6)-C(32)	108.8(19)	N(6)-N(5)-C(31)	112.1(7)
		N(6)-C(32)-C(31)	109.6(17)	N(6)-N(7)-C(32)	108.9(7)
		N(6)-C(32)-C(33)	124(2)	N(7)-C(32)-C(33)	121.7(9)
		N(6)-N(5)-N(4)	105.9(18)	N(7)-N(6)-N(5)	105.8(7)
		O(1)-C(13)-C(12)	109.7(17)		
		O(1)-C(33)-C(32)	112.8(18)		
				N(8)-C(33)-C(32)	117.0(8)

Table S 2. Percentage contributions to the Hirshfeld surface of **3**, **7** and **9**.

	Included surface area		
Contents	**3**	**7**	**9**
H···H	60.3	49.7	62.6
H···N/N···H	21.2	23.6	17.4
H···C/C···H	13.2	19.3	12.9
C···C	2.5	4.3	2.9
N···C/C···N	2.7	1.4	1.7
H···O/O···H		1.6	1.4
N···N			1.1

Table S 3. Comaprison of LODs for different electrochemical sensor for Cd^{2+}, Pb^{2+} and Cu^{2+} determination.

Modified Electrode	LOD {Cd^{2+}}	LOD {Pb^{2+}}	LOD {Cu^{2+}}	[Ref]
Ferrocenyl-Trizol 3@SPCE	9.2 nM	6.3 nM	20.1 nM	This work
Ferrocenyl-Trizol 6@SPCE	30.5 nM	8.7 nM	23.9 nM	This work
Ferrocenyl-Trizol 7@SPCE	4.3 nM	29.0 nM	7.1 nM	This work
Ferrocenyl-Trizol 3@SPCE	3.7 nM	32.0 nM	7.1 nM	This work
Fc-NH2-Ni-MOF	7.1 nM	0.2 nM	6.3 nM	1
trGNO/Fc-NH2-UiO-66.	8.5 nM	0.6 nM	0.8 nM	2
RGO-CS/PLL/GCE	10.0 nM	20.0 nM	20.0 nM	3
NMC	1500 nM	50 nM	–	4
ZJU-27/GCE	1.66 nM	1.1 nM	–	5
GAs-UiO-66-NH_2/GCE	9.0 nM	1.0 nM	8.0 nM	6

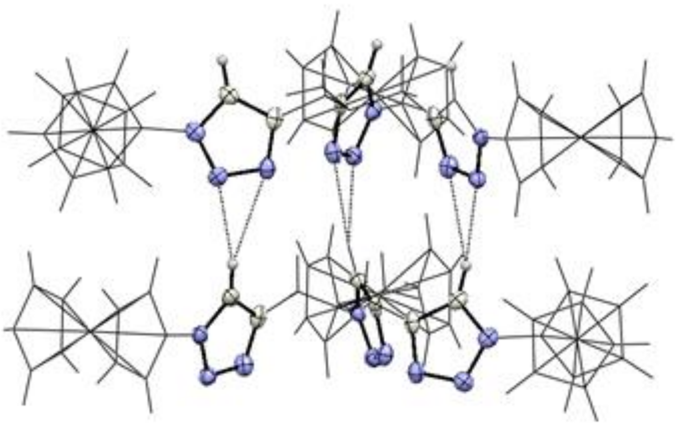

Figure S 1. Illustration of C–H···N (black) contacts in **3**.

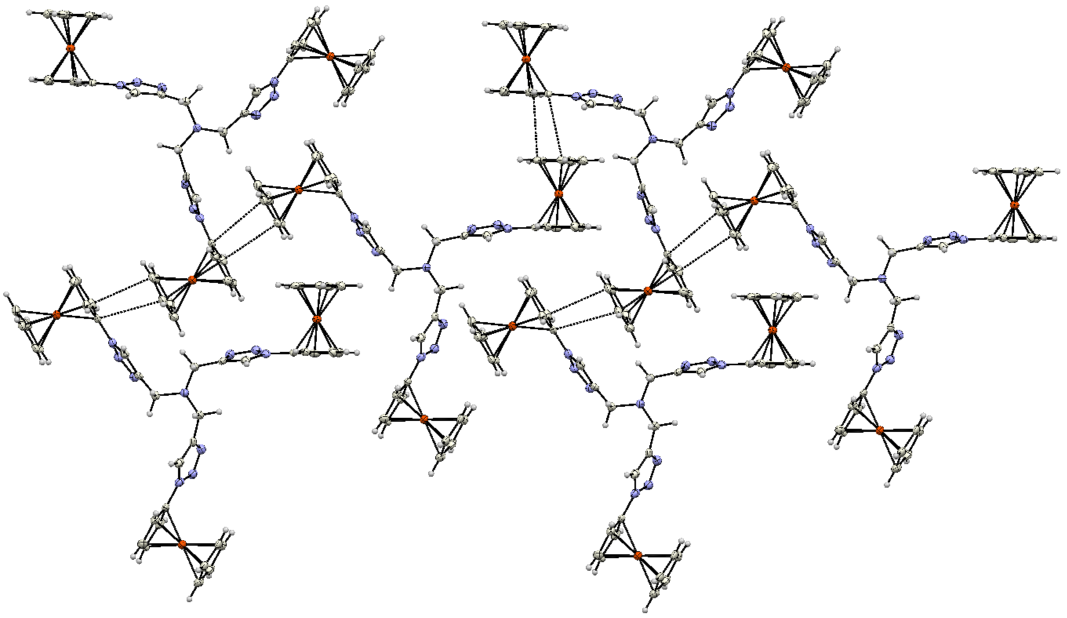

Figure S 2. Illustration of π...π stacking in **3**.

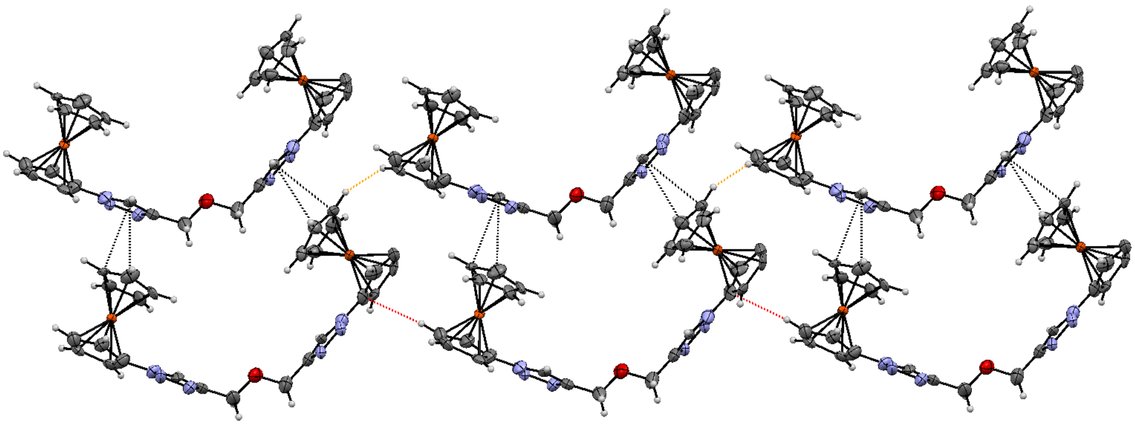

Figure S 3. Illustration of C–H⋯π contacts (red), C–H ⋯ H–C stacking (orange) and π...π stacking (black) in **7**.

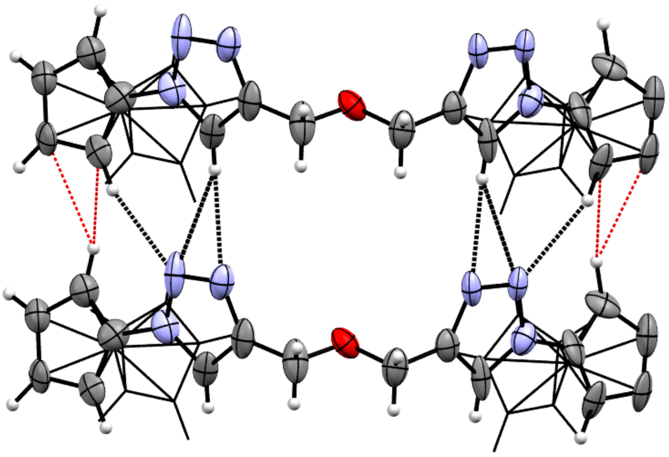

Figure S 4. Illustration of C–H···π (red) and C–H···N (black) contacts in **7**.

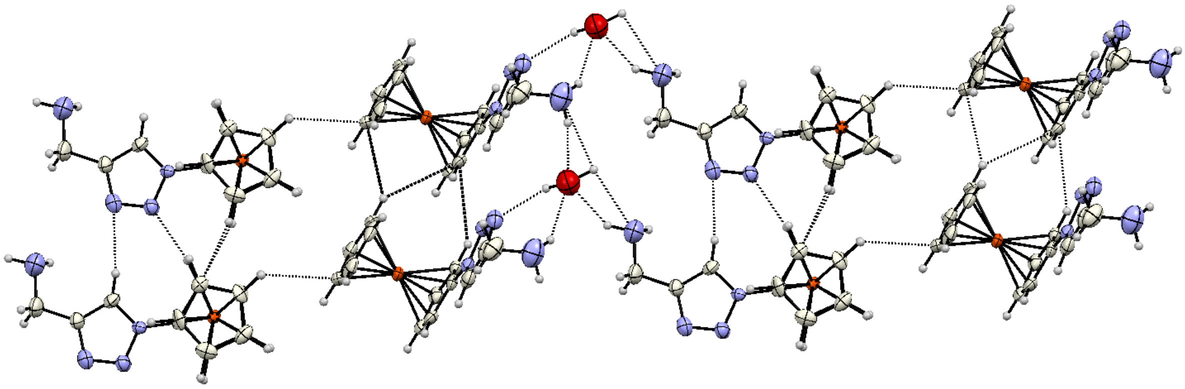

Figure S 5. Intermolecular interaction within the crystal backing of **9**.

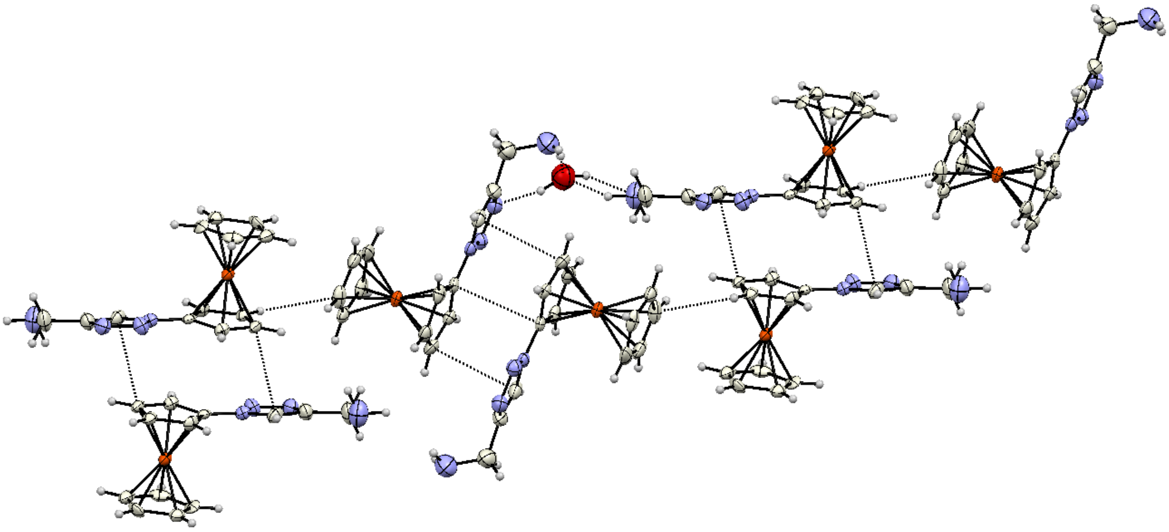

Figure S 6. Intermolecular interaction within the crystal backing of **9**.

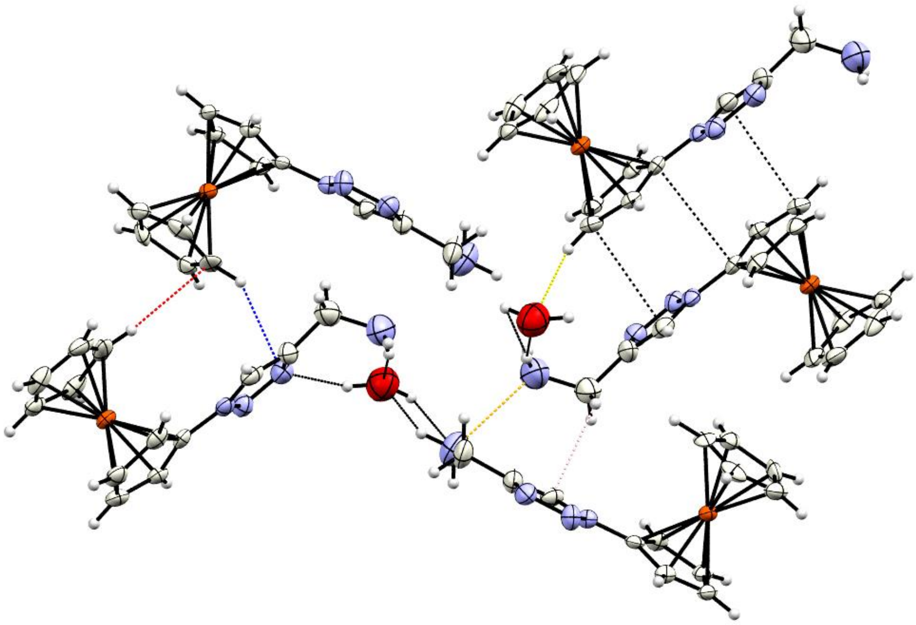

Figure S 7. Illustration of π...π stacking (black), C–H···O contacts (yellow), C–H···C contacts (blue), C–H···C contacts (red), N ··· N contacts (orange) and CH_2···C contacts (pink) in 9.

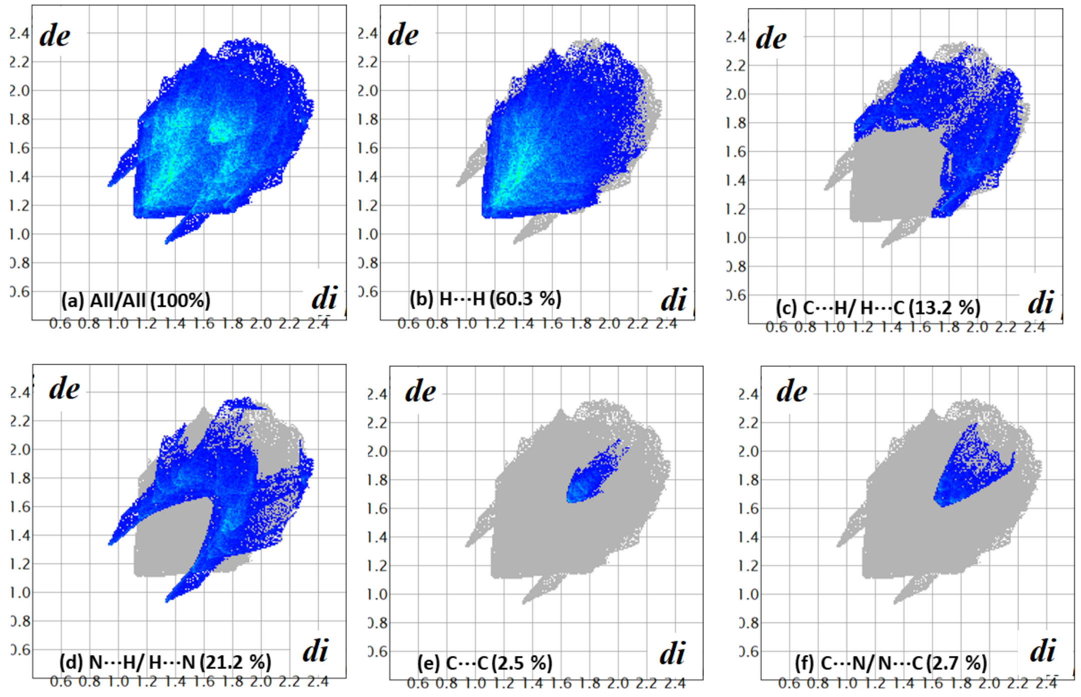

Figure S 8. Full 2-D fingerprint plot for **3**. (a) and the decomposed contacts representing (b) H···H (60.3 %), (c) C···H/H···C (13.2 %), (d) N···H/ H···N (21.2 %), (e) C···C (2.5 %) and (f) C···N/N···C (2.1 %) intermolecular interactions.

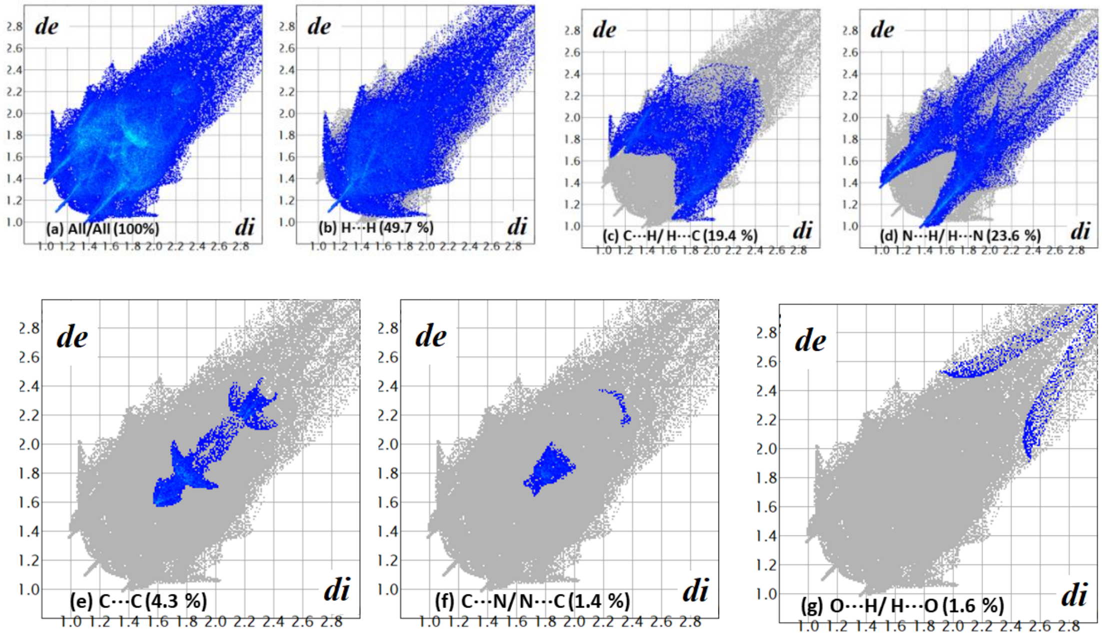

Figure S 9. Full 2-D fingerprint plot for **7**. (a) and the decomposed contacts representing (b) H···H (49.7 %), (c) C···H/H···C (19.4 %), (d) N···H/ H···N (23.6 %), (e) C···C (4.3 %), (f) C···N/N···C (2.1 %), (g) O···H/ H···O (1.6 %) intermolecular interactions.

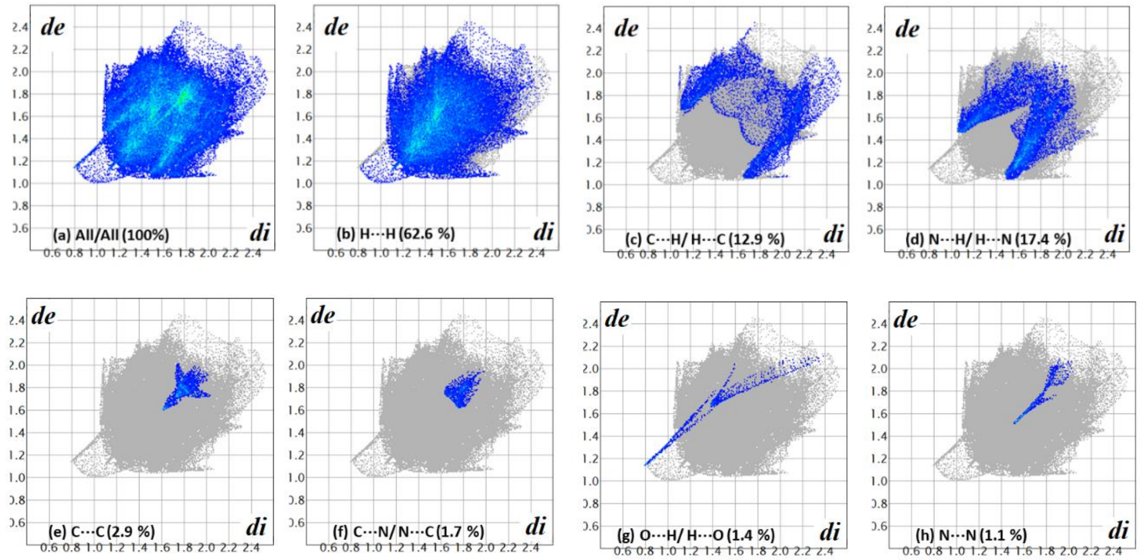

Figure S 10. Full 2-D fingerprint plot for **9**. (a) and the decomposed contacts representing (b) H···H (62.6 %), (c) C···H/H···C (12.9 %), (d) N···H/ H···N (17.4 %), (e) C···C (2.9 %), (f) C···N/N···C (1.7 %), (g) O···H/ H···O (1.4 %) and (g) N···N (1.1 %) intermolecular interactions.

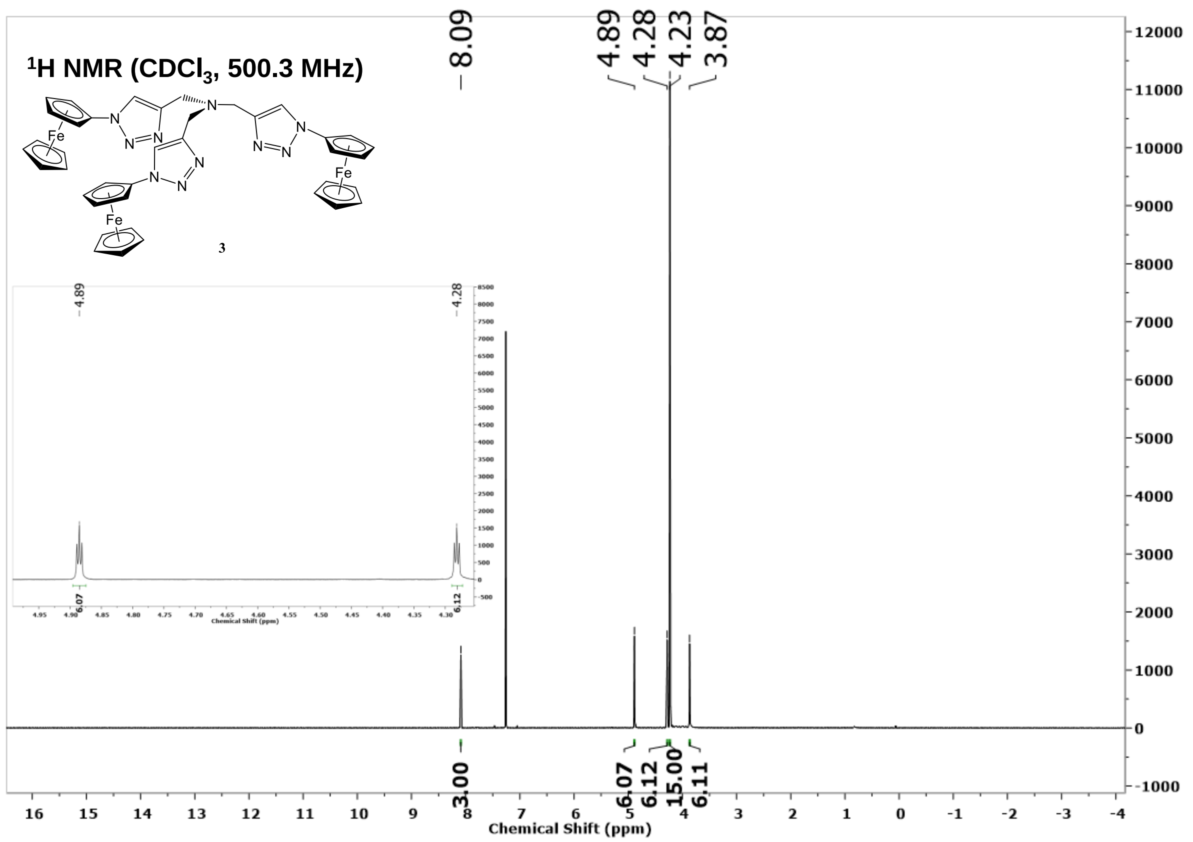

Figure S 11. ^{1}H NMR spectrum of **3** in $CDCl_3$.

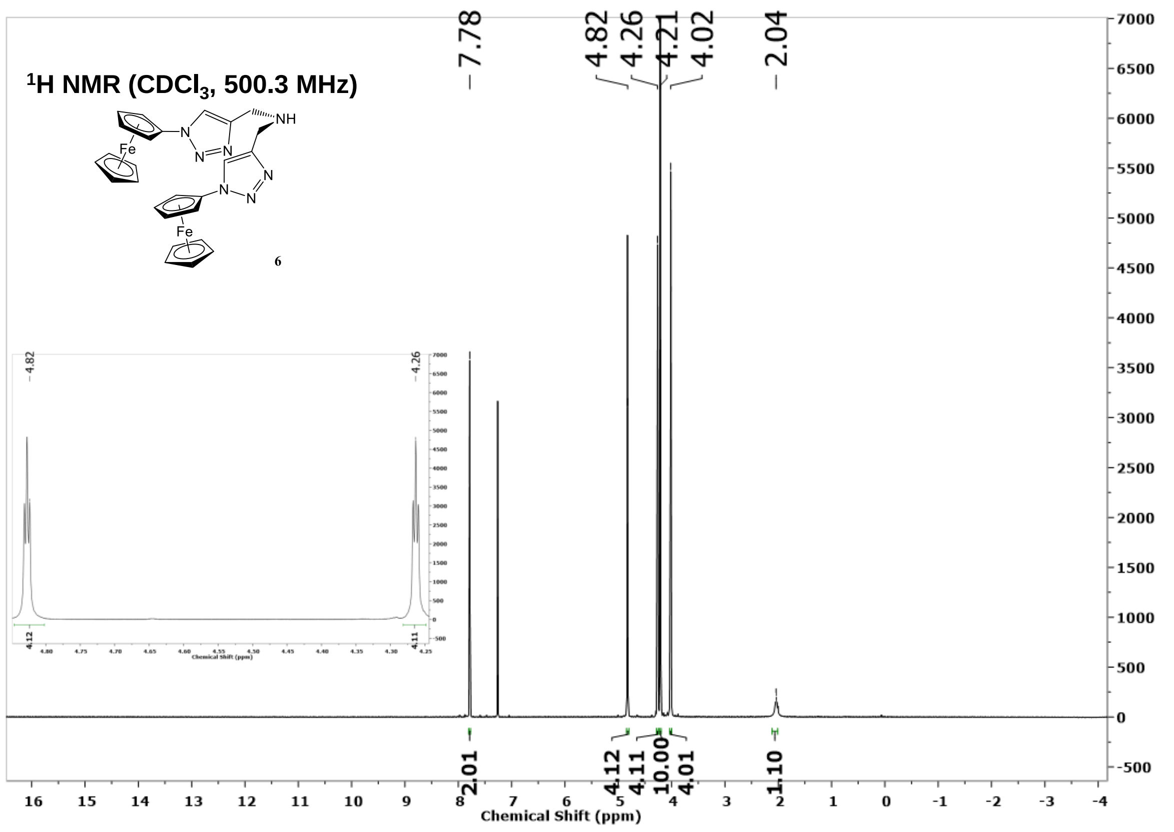

Figure S 12. ^{1}H NMR spectrum of **6** in $CDCl_3$.

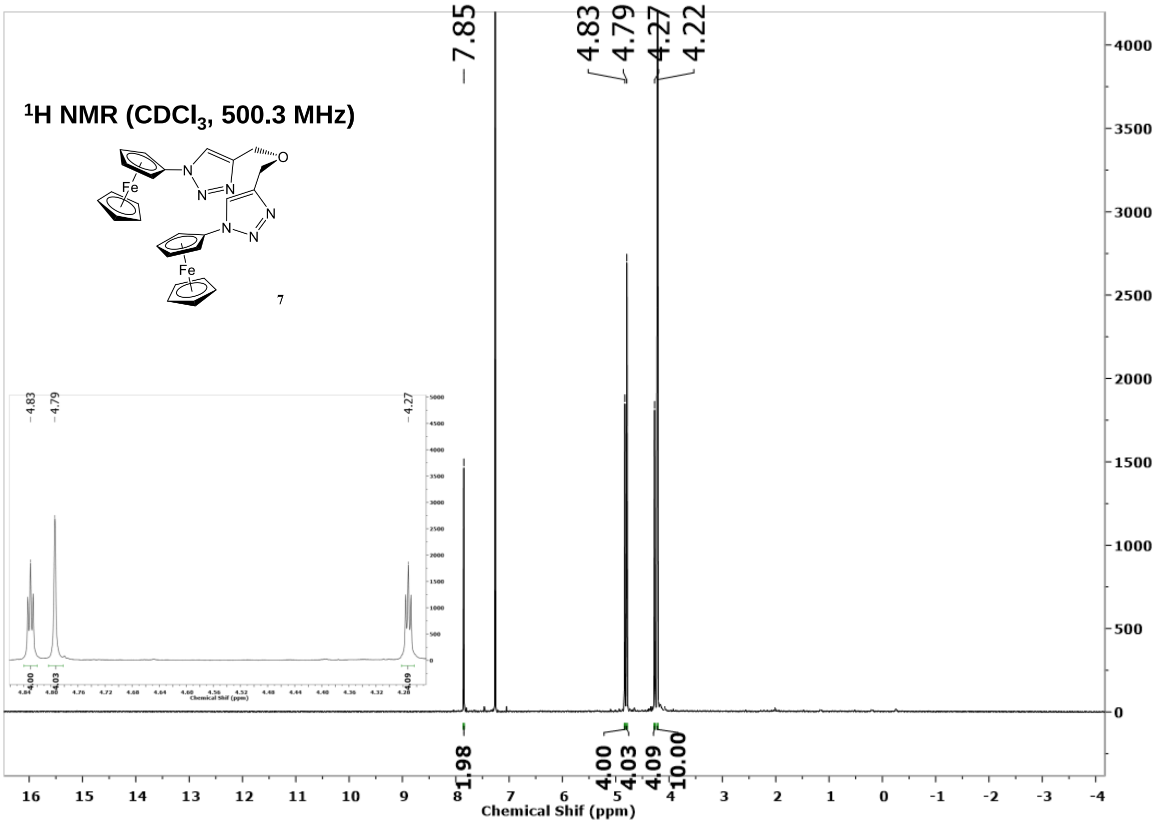

Figure S 13. ^{1}H NMR spectrum of **7** in $CDCl_3$.

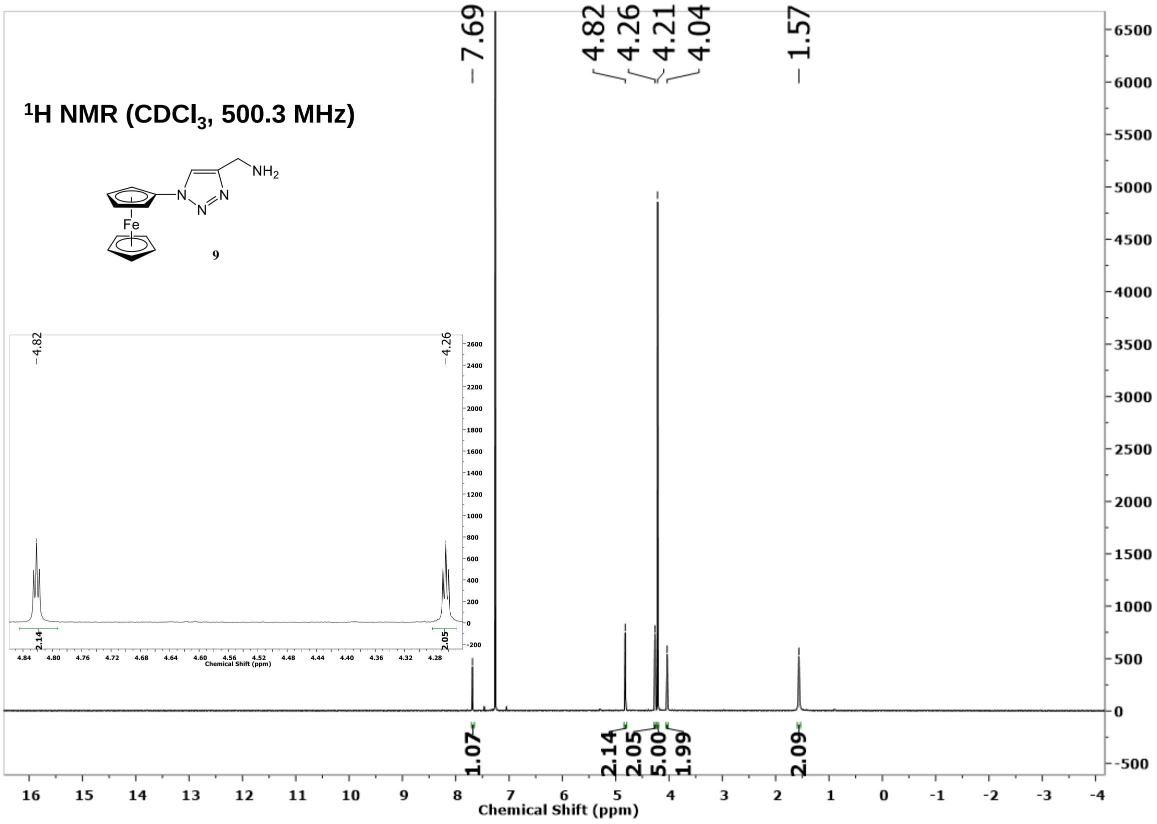

Figure S 14. ^{1}H NMR spectrum of **9** in $CDCl_3$.

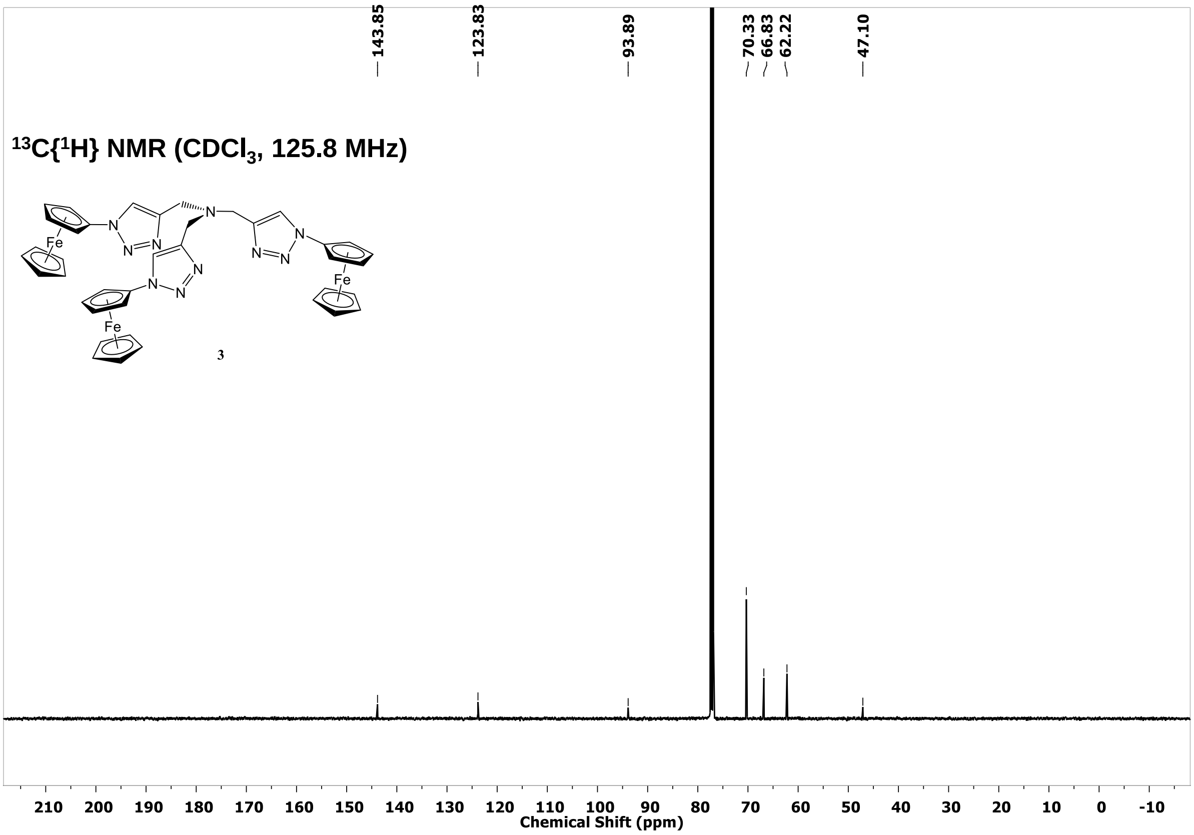

Figure S 15. ^{13}C NMR spectrum of **3** in CDCl₃.

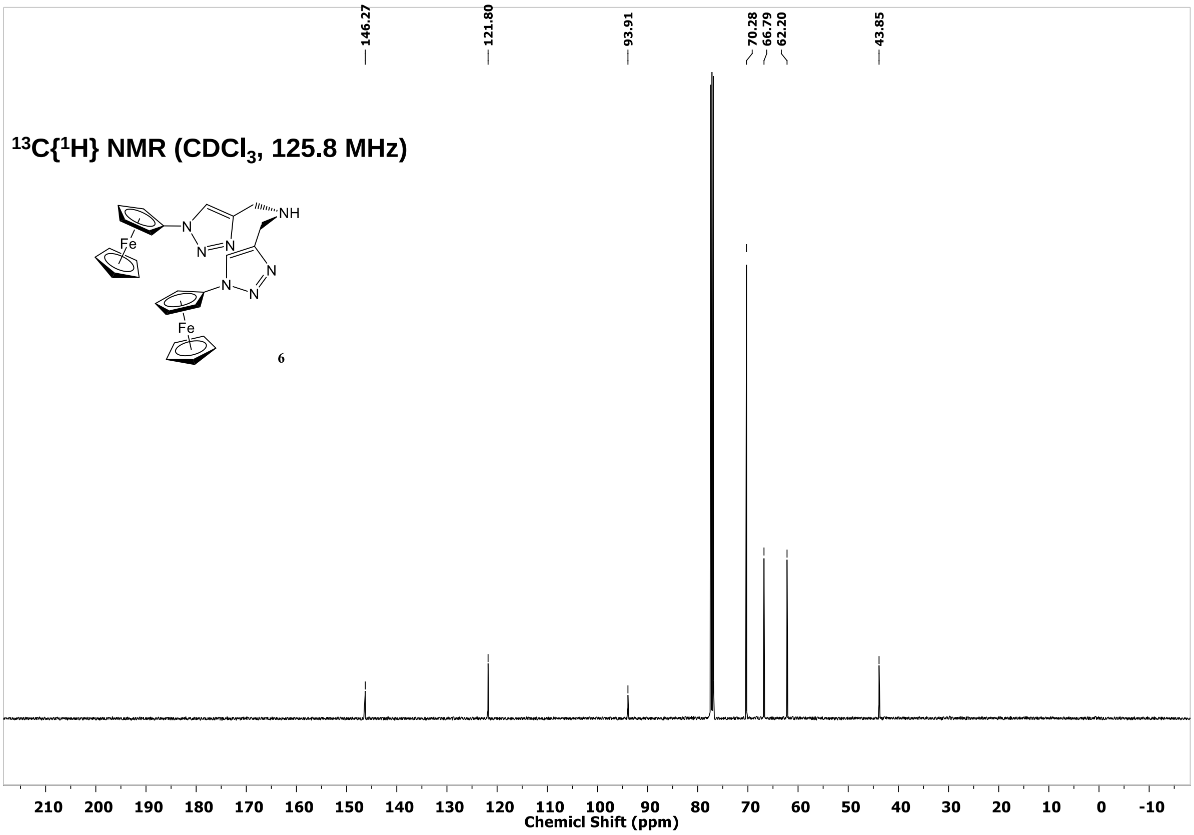

Figure S 16. ^{13}C NMR spectrum of **6** in CDCl₃.

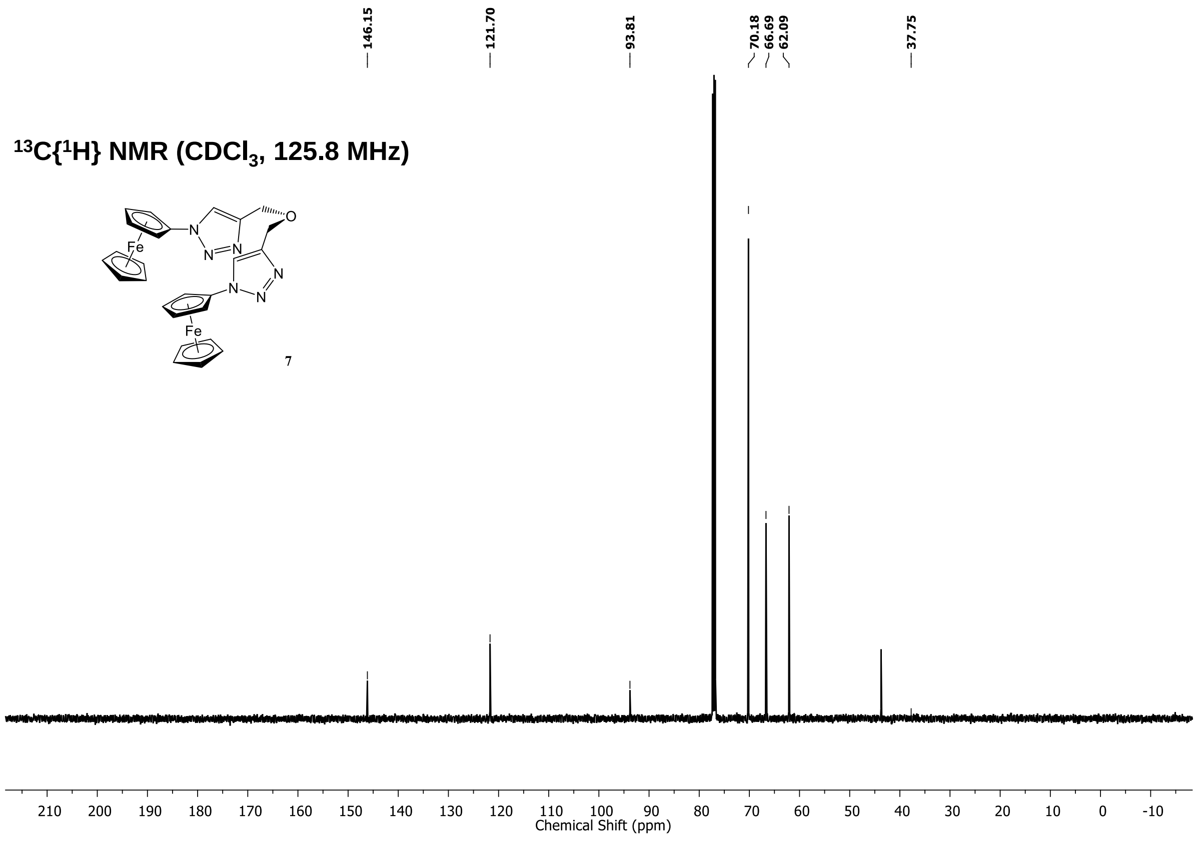

Figure S 17. ^{13}C NMR spectrum of **7** in $CDCl_3$.

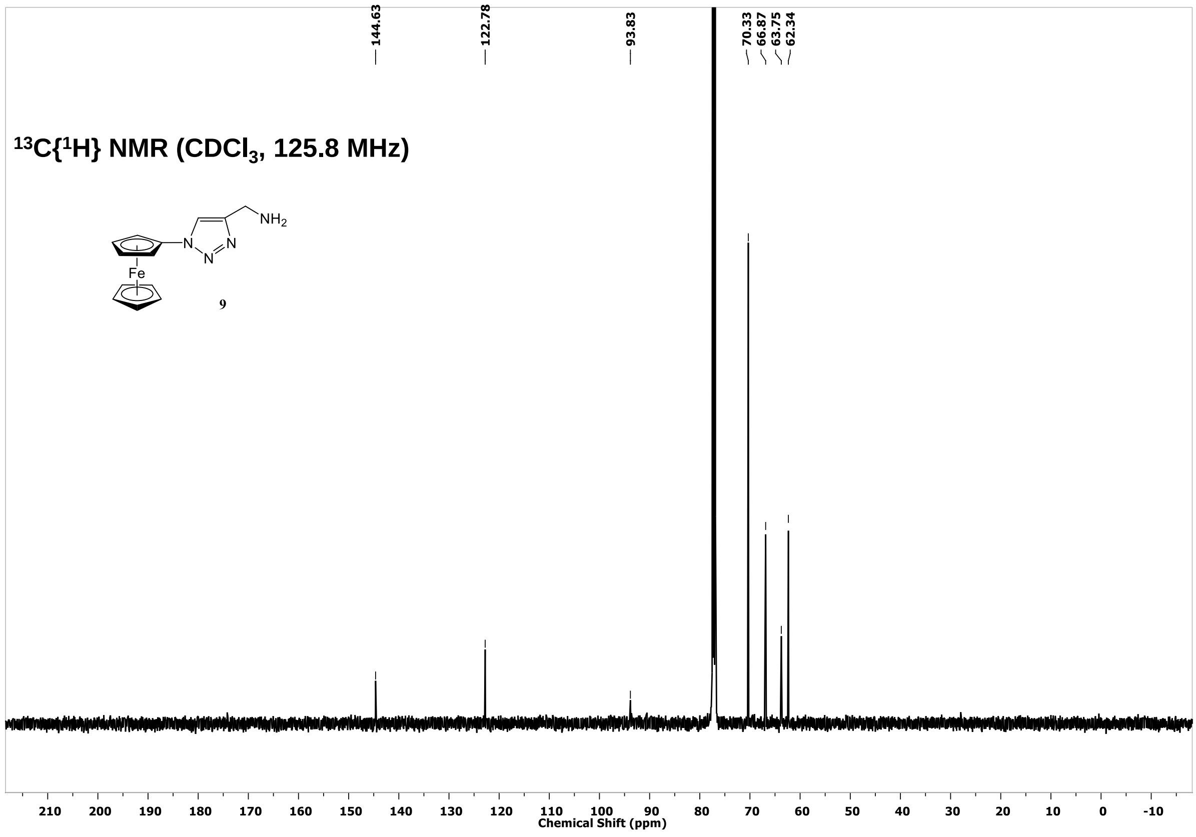

Figure S 18. ^{13}C NMR spectrum of **9** in $CDCl_3$.

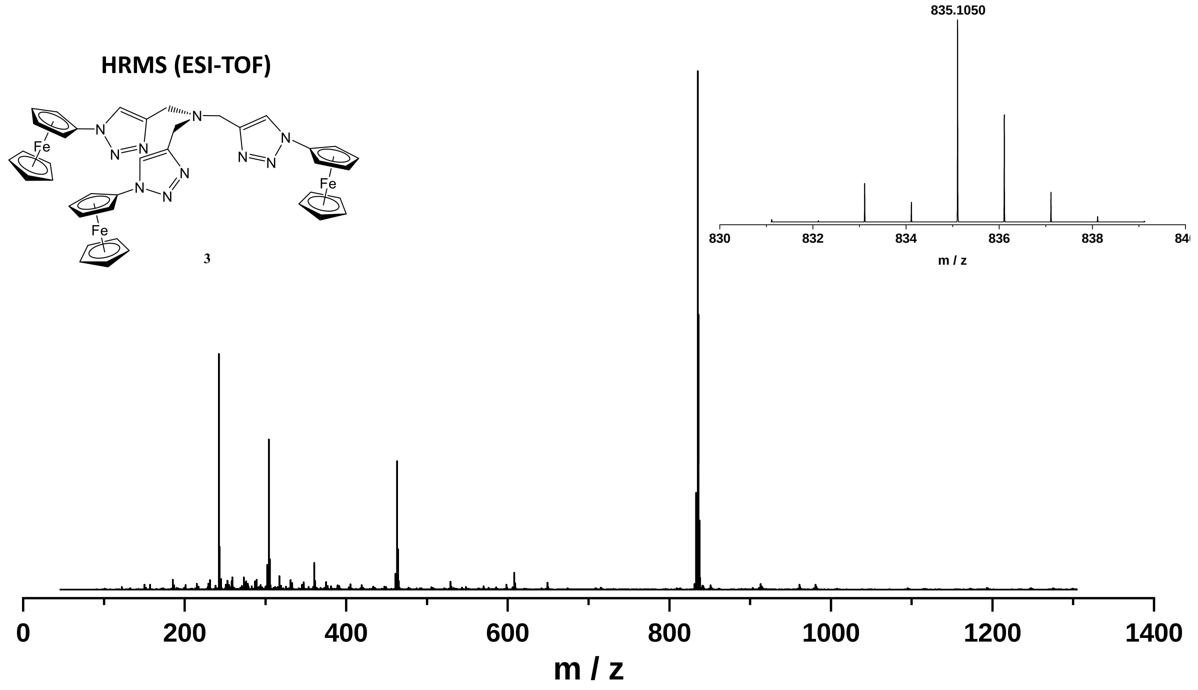

Figure S 19. ESI-TOF Mass spectrum of **3**.

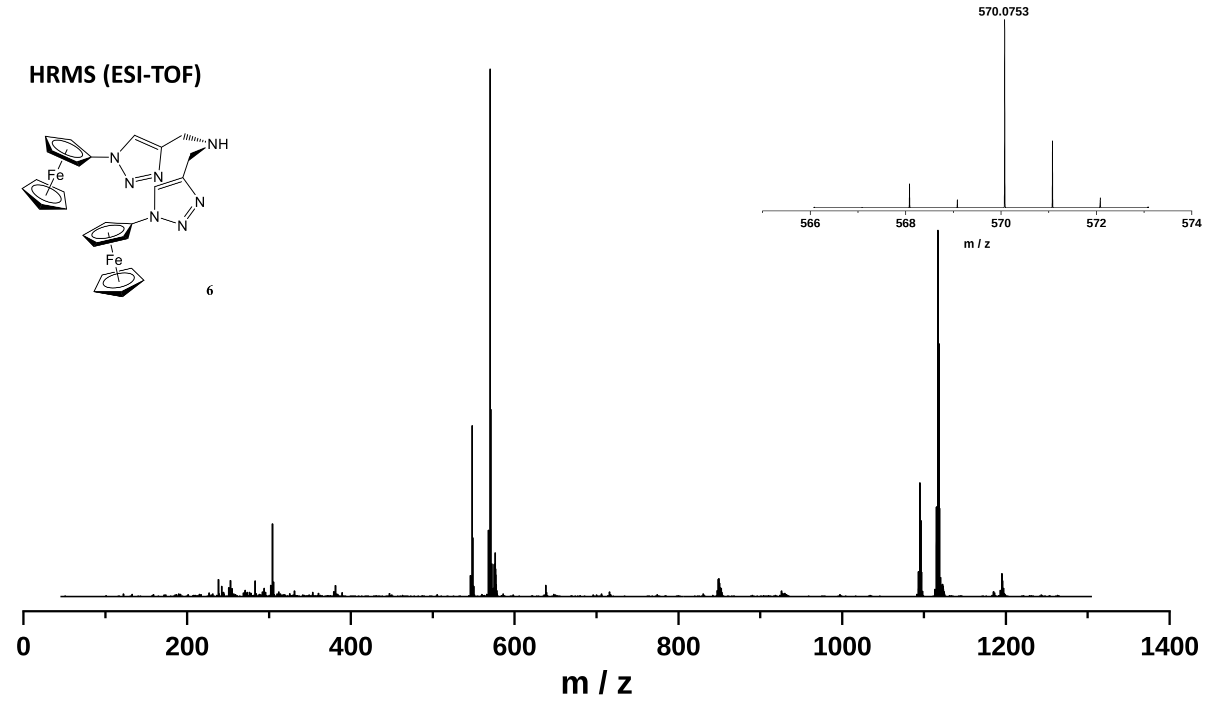

Figure S 20. ESI-TOF Mass spectrum of **6**.

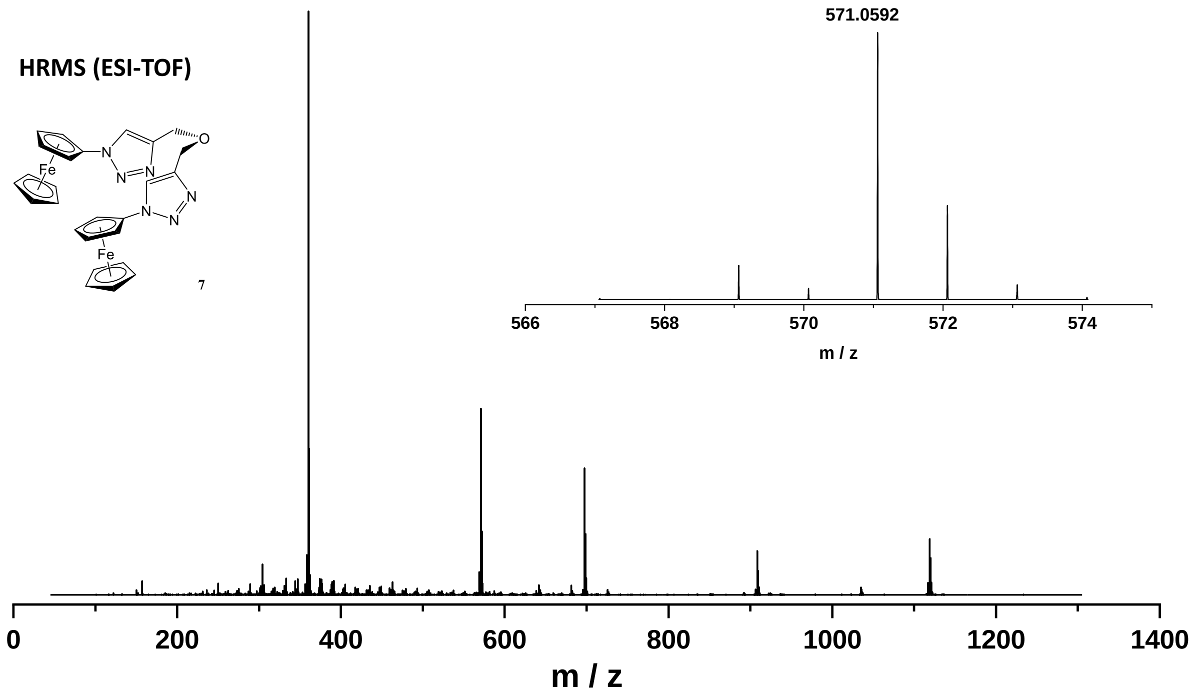

Figure S 21. ESI-TOF Mass spectrum of **7**.

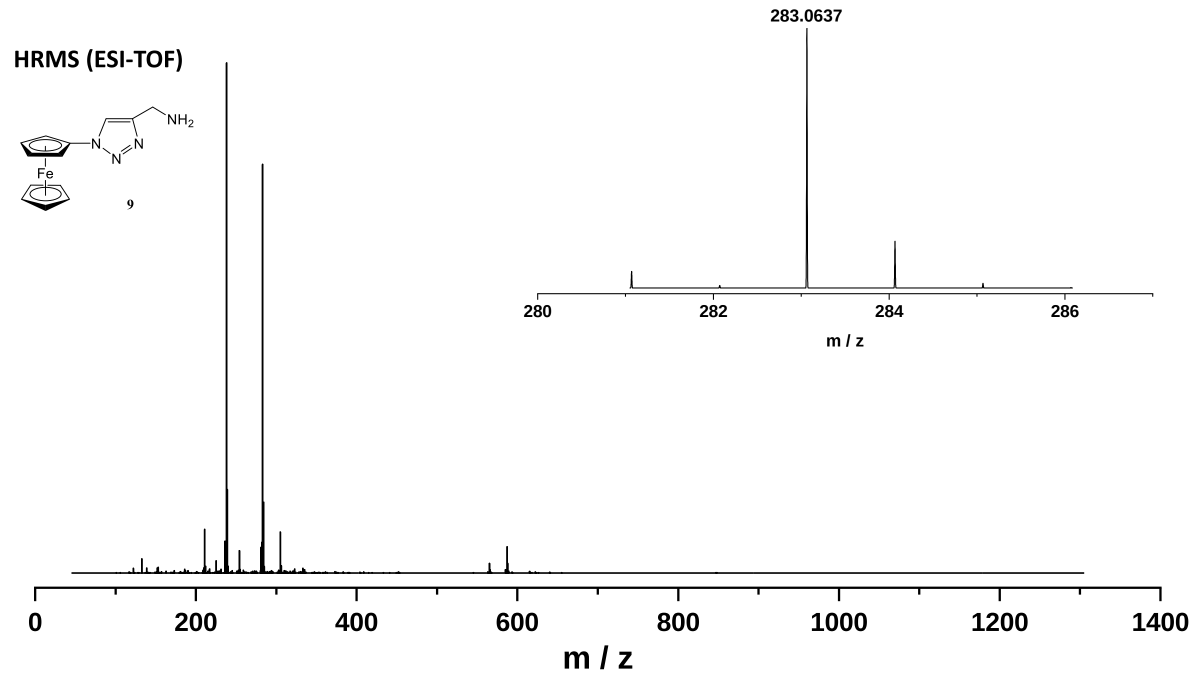

Figure S 22. ESI-TOF Mass spectrum of **9**.

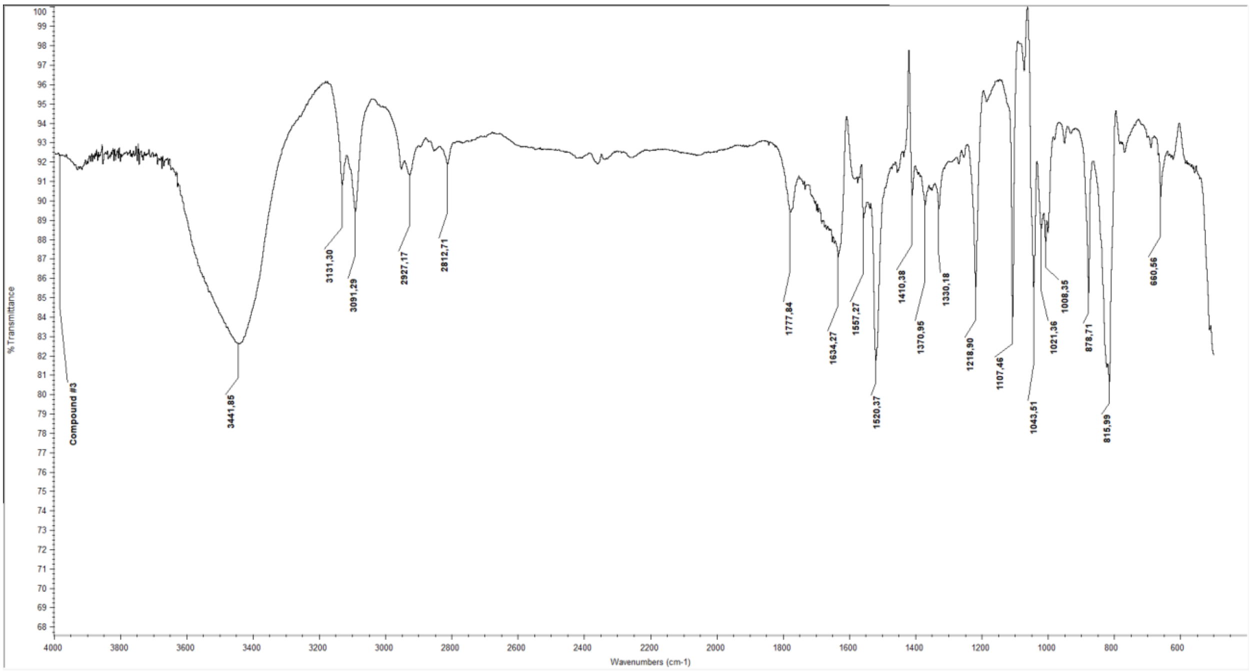

Figure S 23. IR spectrum of **3**.

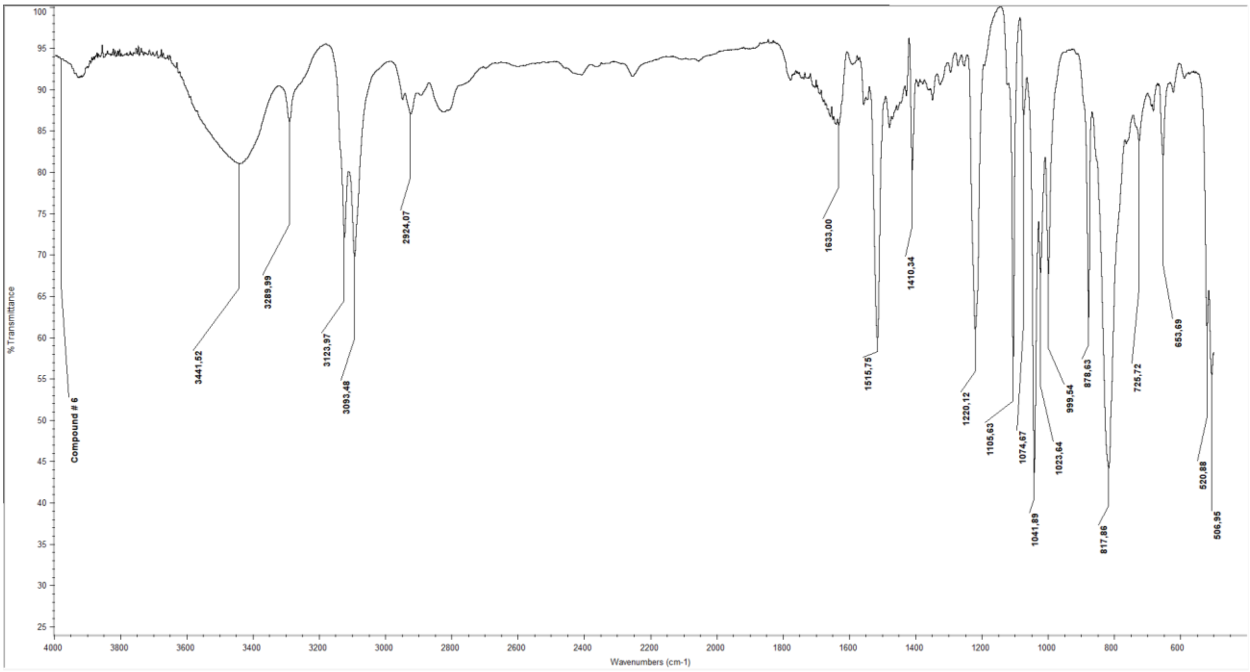

Figure S 24. IR spectrum of **6**.

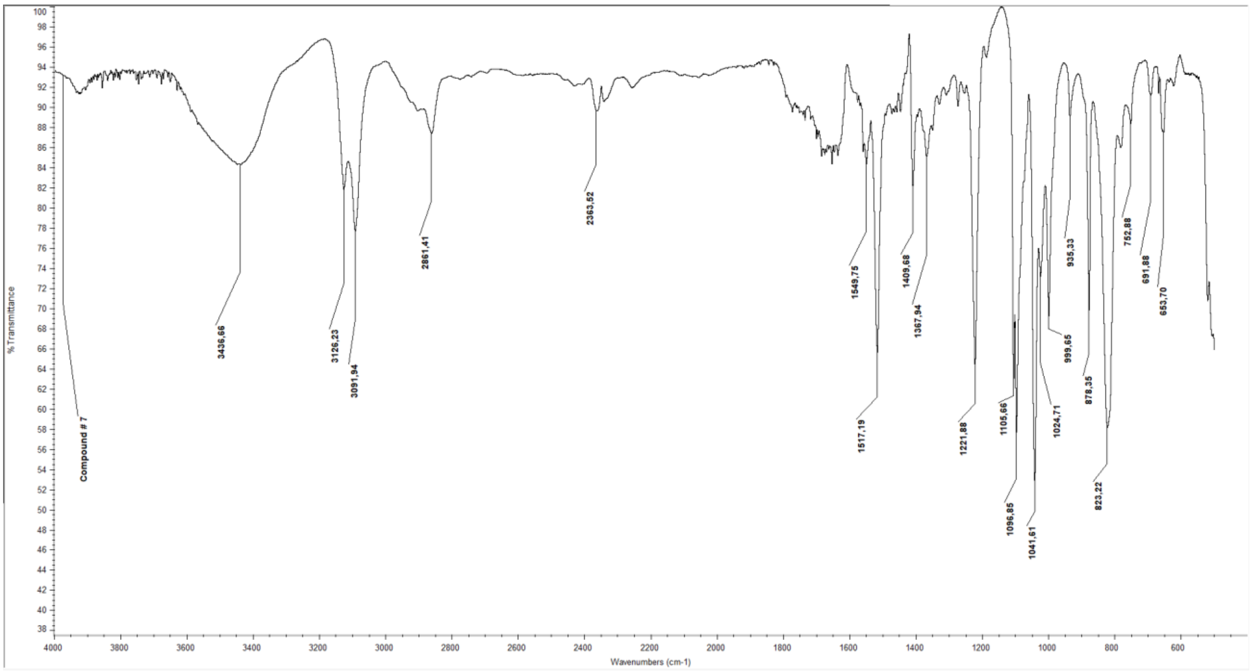

Figure S 25. IR spectrum of **7**.

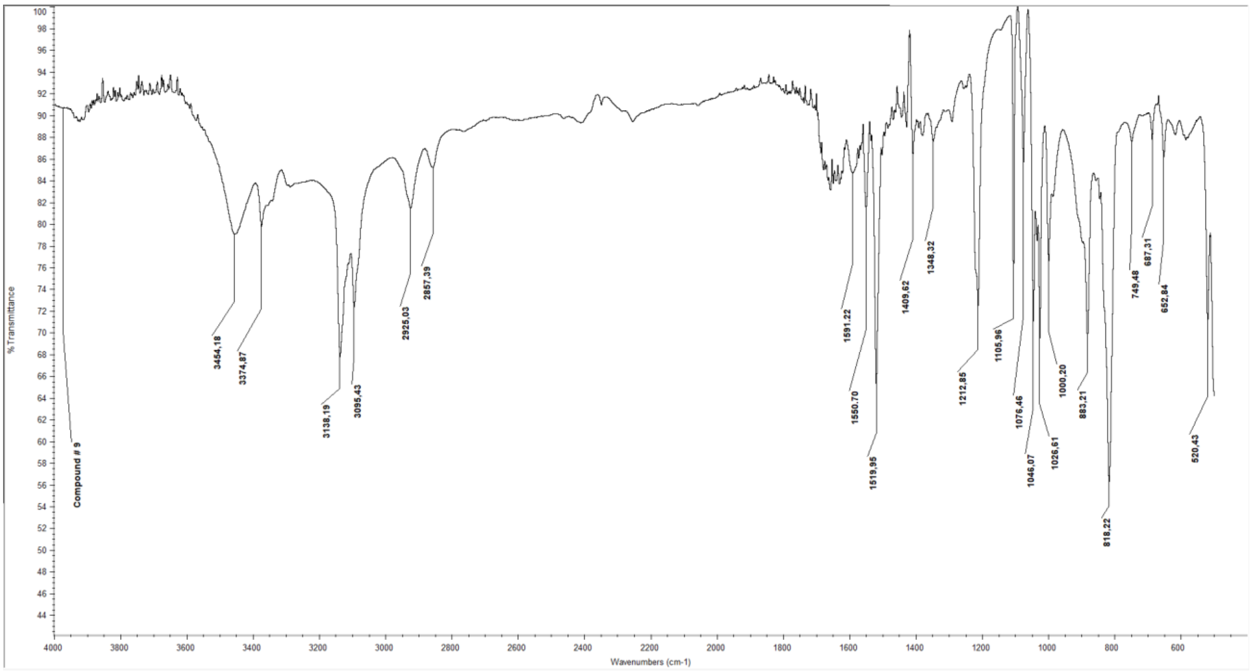

Figure S 26. IR spectrum of **9**.

References

1 J. Wan, Y. Shen, L. Xu, R. Xu, J. Zhang, H. Sun, C. Zhang, C. Yin and X. Wang, *J. Electroanal. Chem.*, 2021, **895**, 115374.

2 X. Wang, Y. Qi, Y. Shen, Y. Yuan, L. Zhang, C. Zhang and Y. Sun, *Sensors Actuators, B Chem.*, 2020, **310**, 127756.

3 Z. Guo, D. di Li, X. ke Luo, Y. hui Li, Q. N. Zhao, M. meng Li, Y. ting Zhao, T. shuai Sun and C. Ma, *J. Colloid Interface Sci.*, 2017, **490**, 11–22.

4 L. Xiao, H. Xu, S. Zhou, T. Song, H. Wang, S. Li, W. Gan and Q. Yuan, *Electrochim. Acta*, 2014, **143**, 143–151.

5 W. Ye, Y. Li, J. Wang, B. Li, Y. Cui, Y. Yang and G. Qian, *J. Solid State Chem.*, 2020, **281**, 121032.

6 M. Lu, Y. Deng, Y. Luo, J. Lv, T. Li, J. Xu, S. W. Chen and J. Wang, *Anal. Chem.*, 2019, **91**, 888–895.
